# Supplementary material for: Rapidly Switchable Universal CAR-T Cells for Treatment of CD123-Positive Leukemia
Source: Mol Ther Oncolytics. 2020 Apr 29;17:408–20. doi: 10.1016/j.omto.2020.04.009 (PMC7240059; doi:10.1016/j.omto.2020.04.009)
Supplement: Document S1. Figures S1–S5 [file mmc1.pdf]

OMTO, Volume *17*

Supplemental Information

Rapidly Switchable Universal CAR-T Cells for Treatment of CD123-Positive Leukemia

Simon Loff, Josephine Dietrich, Jan-Erik Meyer, Julia Riewaldt, Johannes Spehr, Malte von Bonin, Cordula Gründer, Mridula Swayampakula, Kristin Franke, Anja Feldmann, Michael Bachmann, Gerhard Ehninger, Armin Ehninger, and Marc Cartellieri

Figure S1

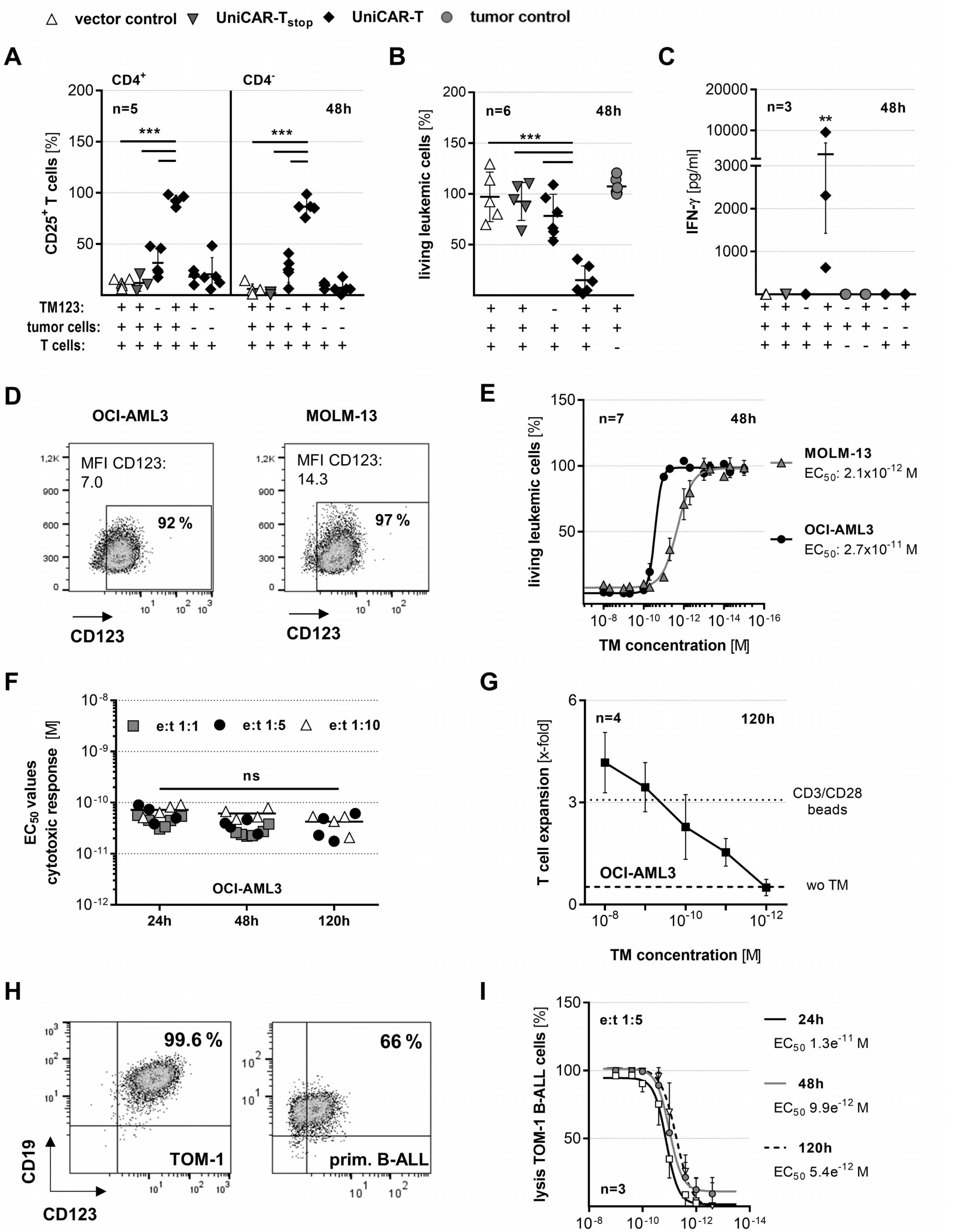
vector control
UniCAR-Tstop
UniCAR-T
tumor control
A
CD4+
CD4-
n=5
48h
***
CD25+ T cells [%]
TM123:
tumor cells:
T cells:
B
n=6
48h
***
living leukemic cells [%]
C
n=3
48h
**
IFN-γ [pg/ml]
D
OCI-AML3
MOLM-13
MFI CD123: 7.0
92 %
MFI CD123: 14.3
97 %
CD123
E
n=7
48h
living leukemic cells [%]
MOLM-13
EC50: 2.1x10-12 M
OCI-AML3
EC50: 2.7x10-11 M
TM concentration [M]
F
e:t 1:1
e:t 1:5
e:t 1:10
ns
EC50 values cytotoxic response [M]
OCI-AML3
24h
48h
120h
G
n=4
120h
T cell expansion [x-fold]
CD3/CD28 beads
OCI-AML3
wo TM
TM concentration [M]
H
99.6 %
TOM-1
66 %
prim. B-ALL
CD19
CD123
I
e:t 1:5
n=3
lysis TOM-1 B-ALL cells [%]
24h
EC50 1.3e-11 M
48h
EC50 9.9e-12 M
120h
EC50 5.4e-12 M

Figure S2

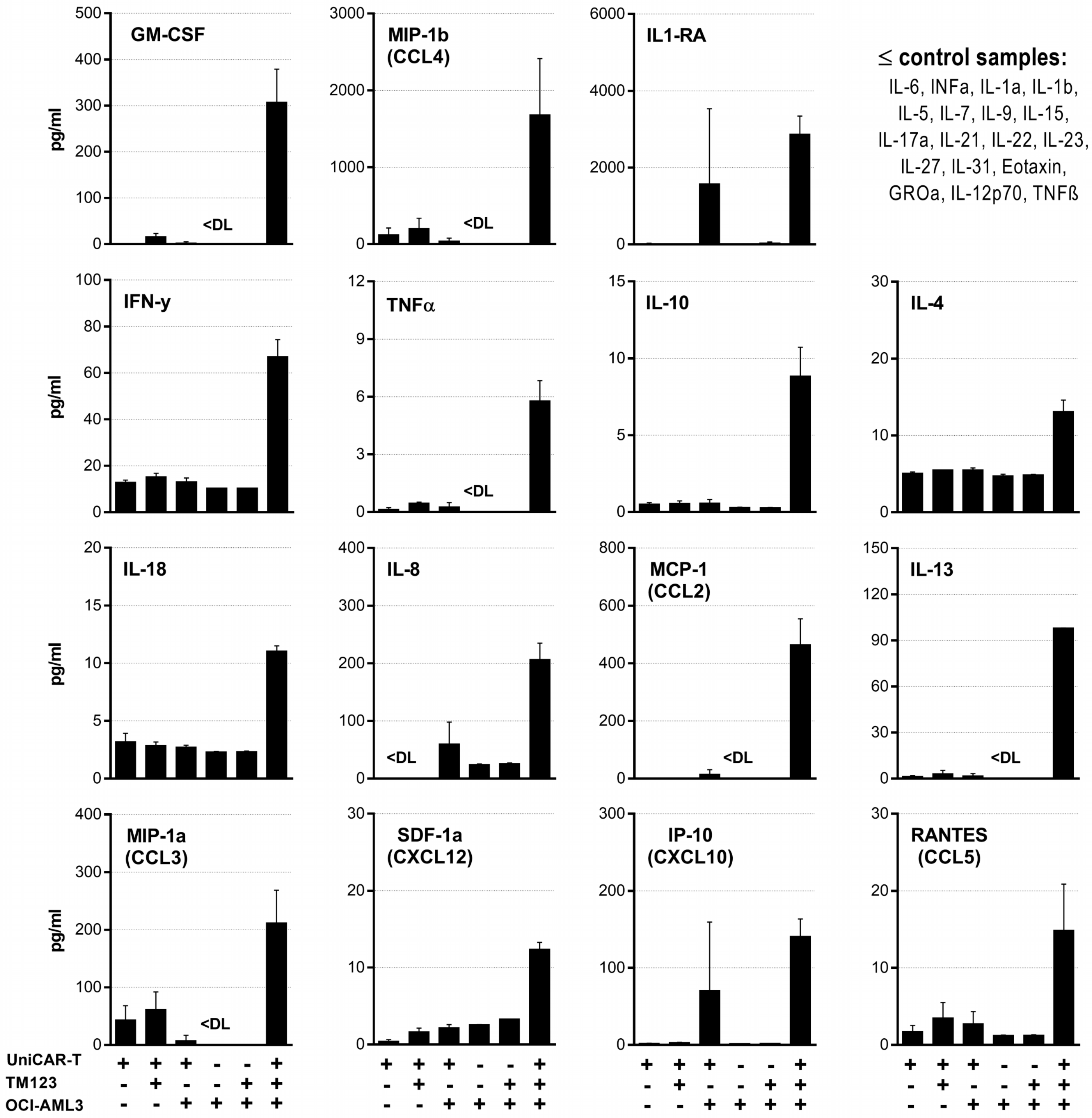
GM-CSF
MIP-1b (CCL4)
IL1-RA
≤ control samples:
IL-6, INFa, IL-1a, IL-1b, IL-5, IL-7, IL-9, IL-15, IL-17a, IL-21, IL-22, IL-23, IL-27, IL-31, Eotaxin, GROa, IL-12p70, TNFß
IFN-y
TNFα
IL-10
IL-4
IL-18
IL-8
MCP-1 (CCL2)
IL-13
MIP-1a (CCL3)
SDF-1a (CXCL12)
IP-10 (CXCL10)
RANTES (CCL5)
pg/ml
<DL
UniCAR-T
TM123
OCI-AML3

Figure S3

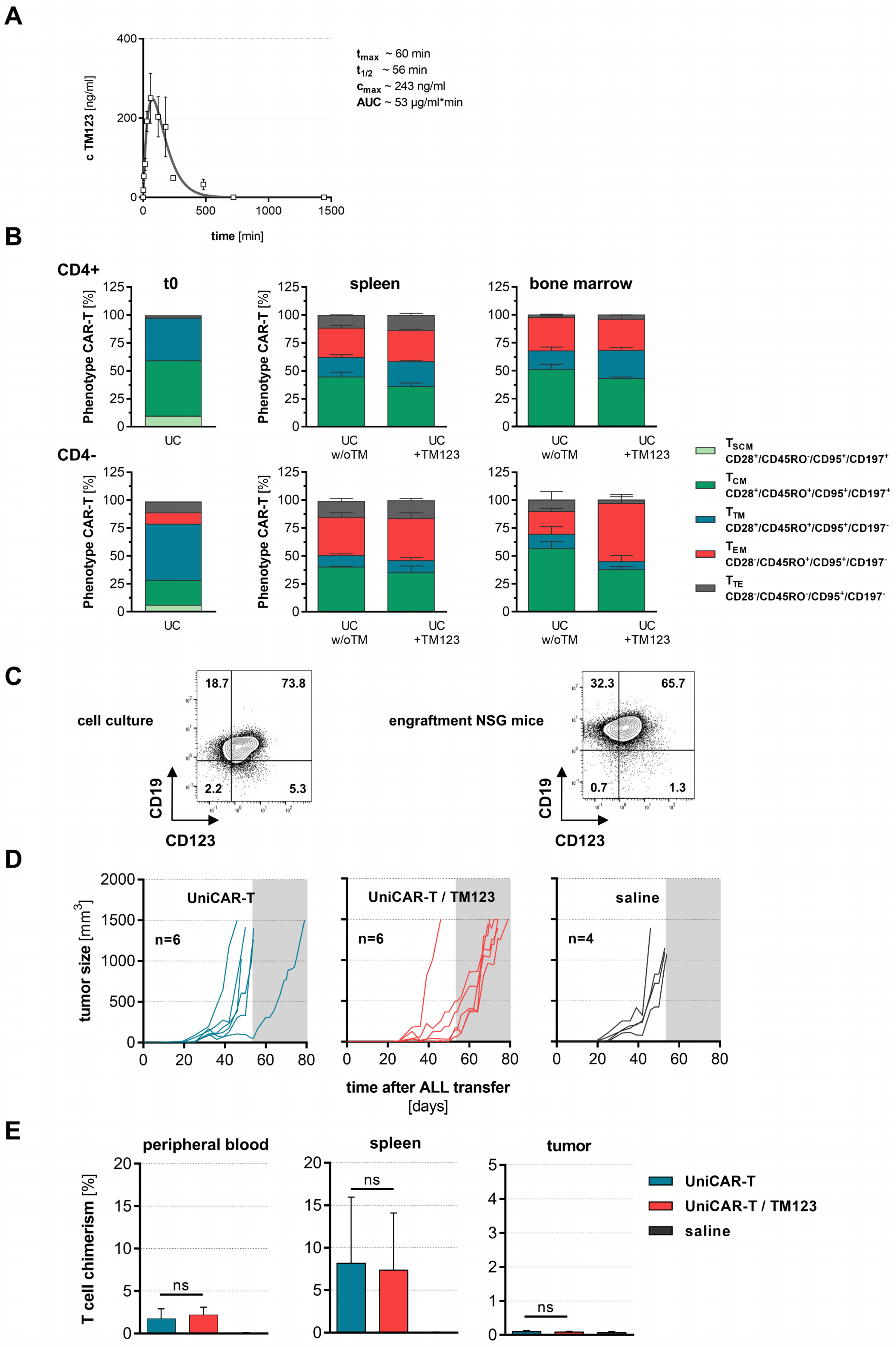
A
c TM123 [ng/ml]
time [min]
t_{max} ~ 60 min
$t_{1/2}$ ~ 56 min
c_{max} ~ 243 ng/ml
AUC ~ 53 µg/ml*min
B
CD4+
CD4-
t0
spleen
bone marrow
Phenotype CAR-T [%]
UC
UC w/oTM
UC +TM123
T_{SCM} CD28+/CD45RO-/CD95+/CD197+
T_{CM} CD28+/CD45RO+/CD95+/CD197+
T_{TM} CD28+/CD45RO+/CD95+/CD197-
T_{EM} CD28-/CD45RO+/CD95+/CD197-
T_{TE} CD28-/CD45RO-/CD95+/CD197-
C
cell culture
18.7
73.8
2.2
5.3
engraftment NSG mice
32.3
65.7
0.7
1.3
CD19
CD123
D
UniCAR-T
UniCAR-T / TM123
saline
n=6
n=6
n=4
tumor size [mm3]
time after ALL transfer [days]
E
peripheral blood
spleen
tumor
T cell chimerism [%]
ns
UniCAR-T
UniCAR-T / TM123
saline

Figure S4

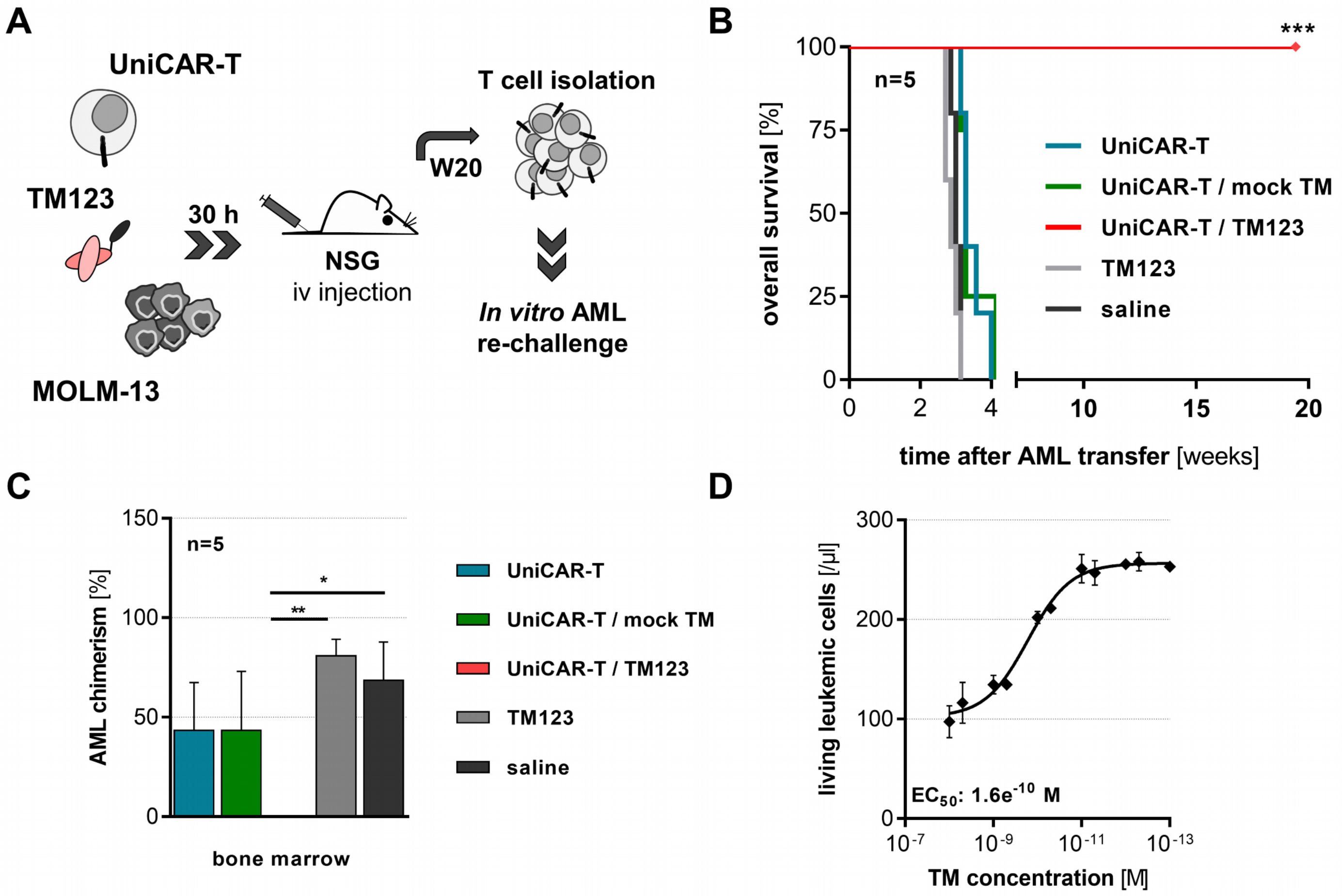
A
UniCAR-T
TM123
MOLM-13
30 h
NSG
iv injection
W20
T cell isolation
In vitro AML
re-challenge
B
***
n=5
overall survival [%]
100
75
50
25
0
0
2
4
10
15
20
time after AML transfer [weeks]
UniCAR-T
UniCAR-T / mock TM
UniCAR-T / TM123
TM123
saline
C
150
100
50
0
n=5
*
**
AML chimerism [%]
bone marrow
UniCAR-T
UniCAR-T / mock TM
UniCAR-T / TM123
TM123
saline
D
300
200
100
0
living leukemic cells [/µl]
EC50: 1.6e-10 M
10-7
10-9
10-11
10-13
TM concentration [M]

Figure S5

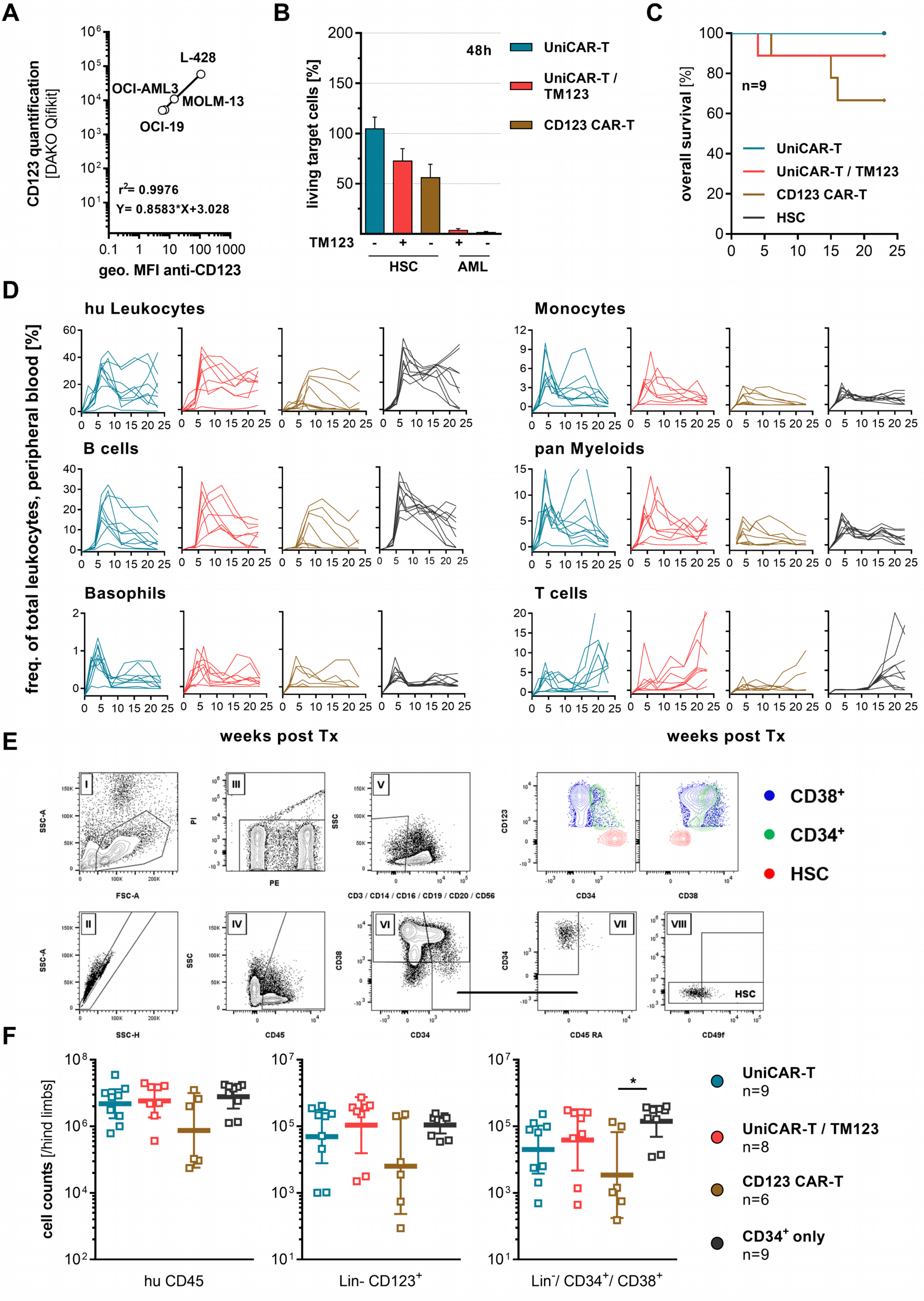
A
CD123 quantification [DAKO Qifikit]
L-428
OCI-AML3
MOLM-13
OCI-19
r²= 0.9976
Y= 0.8583*X+3.028
geo. MFI anti-CD123
B
48h
living target cells [%]
UniCAR-T
UniCAR-T / TM123
CD123 CAR-T
TM123
HSC
AML
C
overall survival [%]
n=9
UniCAR-T
UniCAR-T / TM123
CD123 CAR-T
HSC
D
freq. of total leukocytes, peripheral blood [%]
hu Leukocytes
Monocytes
B cells
pan Myeloids
Basophils
T cells
weeks post Tx
weeks post Tx
E
FSC-A
SSC-A
SSC-H
PE
PI
CD45
SSC
CD3 / CD14 / CD16 / CD19 / CD20 / CD56
CD34
CD38
CD123
CD45 RA
CD49f
HSC
CD38⁺
CD34⁺
HSC
F
cell counts [/hind limbs]
hu CD45
Lin- CD123⁺
Lin⁻/ CD34⁺/ CD38⁺
UniCAR-T
n=9
UniCAR-T / TM123
n=8
CD123 CAR-T
n=6
CD34⁺ only
n=9

Supplemental figure legends

Figure S1. Anti-leukemic response of TM123-redirected UniCAR-T. Human T cells were genetically engineered to express either functional UniCARs, signalling-deficient UniCARs ($UniCAR_{stop}$) or enhanced green fluorescent protein only (vector control). (**A**) Induction of the activation marker CD25 on $CD4^+$ and $CD4^-$ UniCAR-T ($2x10^4$) cultivated with MOLM-13 at an e:t ratio of 1:1 was determined by flow cytometry after 48 h (mean ± SD). (**B**) Lysis of MOLM-13 cells after 48 h in presence of TM123 normalized to controls lacking UniCAR-T (mean ± SD). (**C**) Release of IFN-γ after 48 h co-cultivation of UniCAR-T with MOLM-13 (mean ± SEM). (**D**) CD123 expression on the AML cell lines OCI-AML3 and MOLM-13. (**E**) EC_{50} values for effective cytotoxic responses of TM123-activated UniCAR-T against OCI-AML3 and MOLM-13 after 48 h cultivation (mean ± SD). (**F**) Cytotoxic responses against OCI-AML-3 ($5x10^4$) mediated by decreasing numbers of UniCAR-T (mean). (**G**) Expansion of UniCAR-T after 120 h cultivation with OCI-AML3 cells at e:t 1:10. UniCAR-induced T cell expansion was compared to expansion-rates upon stimulation with CD3 / CD28 T cell activator beads (mean ± SEM). (**H**) CD123 expression on the B-ALL cell line TOM-1 and primary B-ALL blasts. (**I**) EC_{50} values for effective cytotoxic responses of TM123-activated UniCAR-T against TOM-1 B-ALL cells after 24h, 48h and 120h cultivation (mean ± SD). Data was obtained for indicated numbers of T cell donors. Statistical significance for (**A**), (**B**), and (**F**) was assessed by one-way analysis of variance (ANOVA) with *post hoc* Dunnet's multiple comparison test (* $P \leq 0.05$, ** $P \leq 0.01$, *** $P \leq 0.001$, ns, non-significant).

Figure S2. Cytokine profile of TM123-redirected UniCAR-T after cultivation with OCI-AML3. UniCAR-T were cultured together with OCI-AML3 cells in an effector to target cell ratio (e:t) of 1:1 in the presence or absence of 10 nM TM123 for 48 h. Cytokine release was quantified from cell-free culture supernatants using a cytokine-chemokine-34plex analysis. Data was obtained for n=2 independent T cell donors (mean ± deviation).

Figure S3. *In vivo* efficacy of TM123-redirected UniCAR-T against systemic and extramedullary acute leukemia. (**A**) In order to determine half-life of TM123, NSG mice were injected intraperitoneally (ip) with 1000 ng/g TM123, normalized to the bodyweight of mice. Peripheral blood samples were taken at indicated time points. Concentration of TM123 was determined via in-house ELISA.[30] (**B**) Analysis of the UniCAR-T phenotype after the TM123 treatment cycle in the systemic MOLM-13 xenograft model was performed via staining of CD3, CD4, CD28, CD45RO, CD95 and CD197. Resulting T cell subsets were classified as stem cell memory T cells (T_{SCM}), central memory T cells (T_{CM}), transitional memory T cells (T_{TM}), effector memory T cells (T_{EM}) and terminal effector T cells (T_{TE}) (mean ± SEM) according to Mahnke et al. 2013.[36] (**C**) Subcutaneously (sc) transplanted primary B-ALL blasts retain stable CD123 surface expression in NSG mice. (**D**) Tumor progression of sc transplanted primary B-ALL monitored for all individual mice. Highlighted regions indicate TM123-free period post tumor-therapy. (**E**) Analysis of $CD3^+$ T cell frequencies in peripheral blood, spleen and tumor tissue at the individual end points (mean ± SEM). Statistical significance was assessed by nonparametric one-way analysis of variance (ANOVA; Kruskal-Wallis test) with *post hoc* Dunn's multiple comparison test (* $P \leq 0.05$, ns, non-significant).

Figure S4. TM123-redirected UniCAR-T eliminate acute leukemia in an AML-Winn type assay. (**A**) UniCAR-T ($1x10^5$) were pre-incubated with MOLM-13 cells at an effector to target cell (e:t) ratio of 1:1 with or without TM123 for 30 h prior intravenous (iv) transplantation into immunodeficient NSG mice (Winn-assay). Subsequently, isolated T cells were re-challenged with MOLM-13 *in vitro*. (**B**) Overall survival was monitored for 20 weeks. (**C**) Significant reduction of leukemic cells in BM after *ex vivo* pre-incubation (Winn-assay) of MOLM-13 with UniCAR-T and 5 nM TM123. (**D**) After 20 weeks of engraftment in NSG mice purified T cells from spleens were re-challenged with MOLM-13 cells *in vitro*. Anti-tumor response of TM123-redirected UniCAR-T was determined after 48 h at e:t 1:1. Statistical significance was assessed by (**B**) Kaplan-Meier method with log-rank (Mantel-Cox) or by (**C**) nonparametric one-way analysis of variance (ANOVA; Kruskal-Wallis test) with *post hoc* Dunn's multiple comparison test (* $P \leq 0.05$, ** $P \leq 0.01$, *** $P \leq 0.001$).

Figure S5. Analysis of human cell engraftment in peripheral blood and bone marrow of humanized NSG mice. (**A**) CD123 was quantified on representative cell lines using DAKO Qifikit®. CD123 density on further cell lines, primary cells or patient derived samples was calculated via antigen standard curve. (**B**) Cytotoxic response of transplanted donor-specific UniCAR-T in presence or absence of 5 nM TM123 and CD123 CAR-T against isolated $CD34^+$ hematopoietic progenitors or MOLM-13 cells was assessed after 48 h in parallel to the *ex vivo* incubation samples (mean ± SD). (**C**) Overall survival of humanized NSG mice. (**D**) Human leukocyte subsets in the peripheral blood (PB) of all individual recipient mice. (**E**) Gating strategy for identification of HSC-enriched cell pools in bone marrow (BM) samples. (**F**) Absolute numbers of human leukocytes and hematopoietic progenitors per hind limbs (femur + tibia). All samples were analyzed 23 weeks post transplantation. Statistical significance was assessed by parametric one-way analysis of variance (ANOVA) with *post hoc* Dunnet's multiple comparison test (* $P \leq 0.05$).
