# Supplementary material for: Rapidly Switchable Universal CAR-T Cells for Treatment of CD123-Positive Leukemia
Source: Mol Ther Oncolytics. 2020 Apr 29;17:408–20. doi: 10.1016/j.omto.2020.04.009 (PMC7240059; doi:10.1016/j.omto.2020.04.009)
Supplement: Document S2. Article plus Supplemental Information [file mmc2.pdf]

Molecular Therapy
Oncolytics
Original Article

Rapidly Switchable Universal CAR-T Cells for Treatment of CD123-Positive Leukemia

Simon Loff,[1] Josephine Dietrich,[2,8] Jan-Erik Meyer,[2,8] Julia Riewaldt,[2] Johannes Spehr,[2] Malte von Bonin,[3,6] Cordula Gründer,[1] Mridula Swayampakula,[1] Kristin Franke,[1] Anja Feldmann,[5] Michael Bachmann,[4,5,6,7] Gerhard Ehninger,[1,2] Armin Ehninger,[1,2] and Marc Cartellieri[1,2]

[1]GEMoaB Monoclonals GmbH, 01307 Dresden, Germany; [2]Cellex Patient Treatment GmbH, 01307 Dresden, Germany; [3]Medical Clinic and Policlinic I, University Hospital "Carl Gustav Carus," TU Dresden, 01307 Dresden, Germany; [4]University Cancer Center "Carl Gustav Carus," TU Dresden, Tumor Immunology, 01307 Dresden, Germany; [5]Helmholtz-Zentrum Dresden-Rossendorf, Institute of Radiopharmaceutical Cancer Research, 01328 Dresden, Germany; [6]German Cancer Consortium "Carl Gustav Carus," TU Dresden, 01307 Dresden, Germany; [7]National Center for Tumor Diseases, "Carl Gustav Carus," TU Dresden, 01307 Dresden, Germany

Chimeric antigen receptor T cells (CAR-T) targeting CD19 or B cell maturation antigen (BCMA) are highly effective against B cell malignancies. However, application of CAR-T to less differentially expressed targets remains a challenge due to lack of tumor-specific antigens and CAR-T controllability. CD123, a highly promising leukemia target, is expressed not only by leukemic and leukemia-initiating cells, but also by myeloid, hematopoietic progenitor, and certain endothelial cells. Thus, CAR-T lacking fine-tuned control mechanisms pose a high toxicity risk. To extend the CAR-T target landscape and widen the therapeutic window, we adapted our rapidly switchable universal CAR-T platform (UniCAR) to target CD123. UniCAR-T efficiently eradicated CD123$^+$ leukemia *in vitro* and *in vivo*. Activation, cytolytic response, and cytokine release were strictly dependent on the presence of the CD123-specific targeting module (TM123) with comparable efficacy to CD123-specific CAR-T *in vitro*. We further demonstrated a pre-clinical proof of concept for the safety-switch mechanism using a hematotoxicity mouse model wherein TM123-redirected UniCAR-T showed reversible toxicity toward hematopoietic cells compared to CD123 CAR-T. In conclusion, UniCAR-T maintain full anti-leukemic efficacy, while ensuring rapid controllability to improve safety and versatility of CD123-directed immunotherapy. The safety and efficacy of UniCAR-T in combination with TM123 will now be assessed in a phase I clinical trial (ClinicalTrials.gov: NCT04230265).

INTRODUCTION

Despite constant emergence of new treatment options, relapsed or refractory (r/r) malignancies of the hematopoietic system are still associated with poor prognosis. Until today, allogeneic hematopoietic stem cell transplantation (allo-HSCT) is the only curative treatment option for r/r acute myeloid leukemia (AML). However, allo-HSCT is associated with significant treatment-related mortality and morbidity.[1,2] Therefore, innovative new immunotherapeutic concepts have been developed in recent years. The application of monoclonal antibodies (mAbs),[3,4] redirection of autologous T cells with bispecific antibodies,[5] and cellular immunotherapy demonstrated high response rates against r/r hematologic malignancies. In particular *ex vivo* engineered autologous T cells expressing chimeric antigen receptors (CAR-T) against CD19-positive B cell malignancies showed encouraging clinical results with impressive response rates,[6–9] including patients refractory to prior blinatumomab treatment.[10] Such impressive clinical results prompted the US Food and Drug Administration (FDA) and the European Medicines Agency (EMA) to grant marketing authorization to the first CAR-T products.[6,11] Nevertheless, acute adverse events such as treatment-related severe cytokine release syndrome (CRS), neurotoxicity, as well as the development of CD19 CAR-T refractory escape variants in a significant proportion of patients treated limit their therapeutic success.[12–15] Moreover, CAR-T treatment beyond CD19 and B cell maturation antigen (BCMA) remains challenging, as expression of other targets in contrast to the B cell-lineage antigens is less differentiated.

A particularly attractive target for immunotherapy of several hematologic malignancies is CD123, the IL-3 receptor α chain. The high expression levels of CD123 in AML, acute lymphoblastic leukemia (ALL), blastic plasmacytoid dendritic cell neoplasm (BPDCN), hairy cell leukemia, and certain lymphomas[16,17] mark CD123 as an attractive target for CAR-T therapy.[18–21] However, CD123 is also shown to be present on regular cells, including hematopoietic progenitors[4,22–24] and endothelial cells.[25,26] Preclinical studies reported deleterious effects of CD123-directed immunotherapy.[27–29]

Recently, our group developed a rapidly switchable universal CAR-T platform (UniCAR) to allow for a highly controlled and dose-dependent activation of CAR-T.[30] The platform approach was successfully

Received 28 March 2020; accepted 23 April 2020;
https://doi.org/10.1016/j.omto.2020.04.009.

[8]These authors contributed equally to this work.

Correspondence: Marc Cartellieri, Cellex Patient Treatment GmbH, Tatzberg 47, 01307 Dresden, Germany.
E-mail: m.cartellieri@cellex-treatment.me

This is an open access article under the CC BY license (http://creativecommons.org/licenses/by/4.0/).

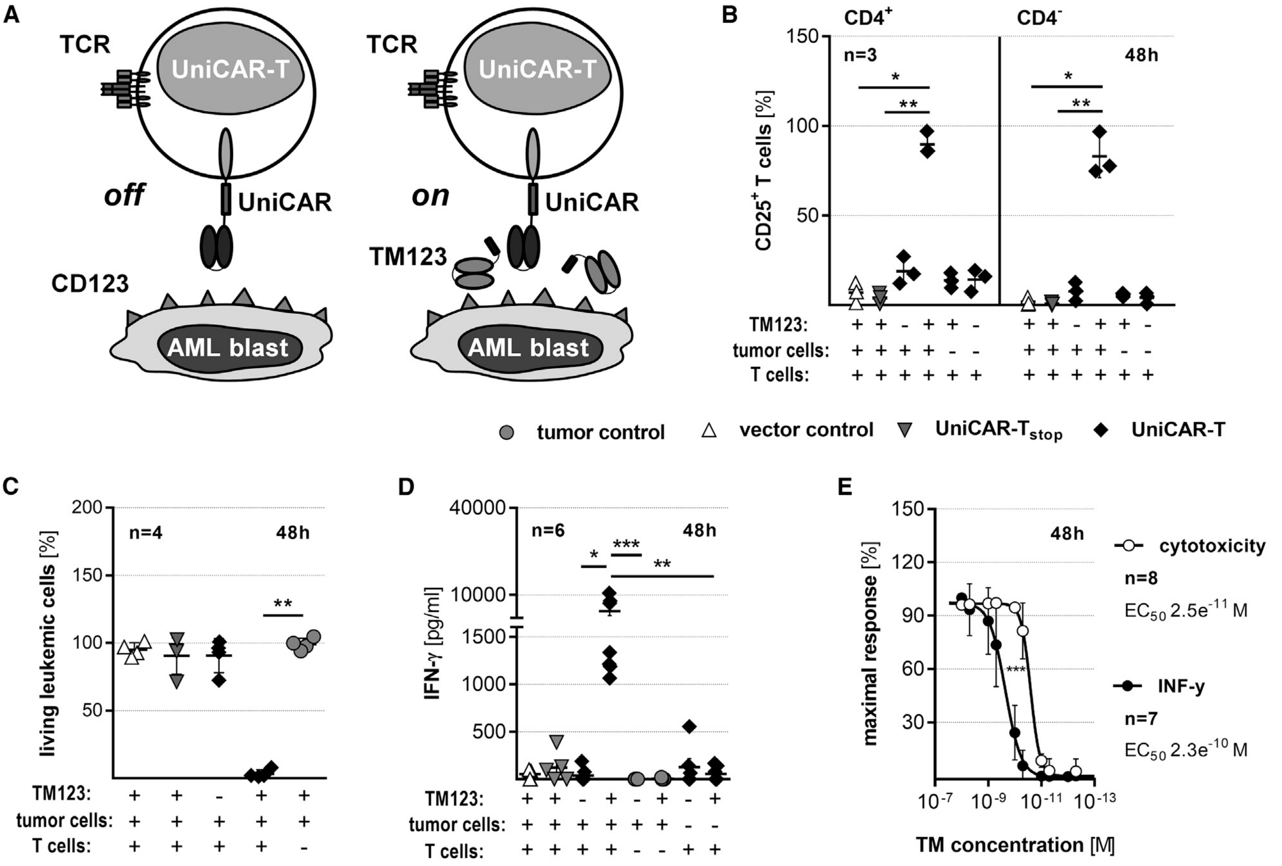

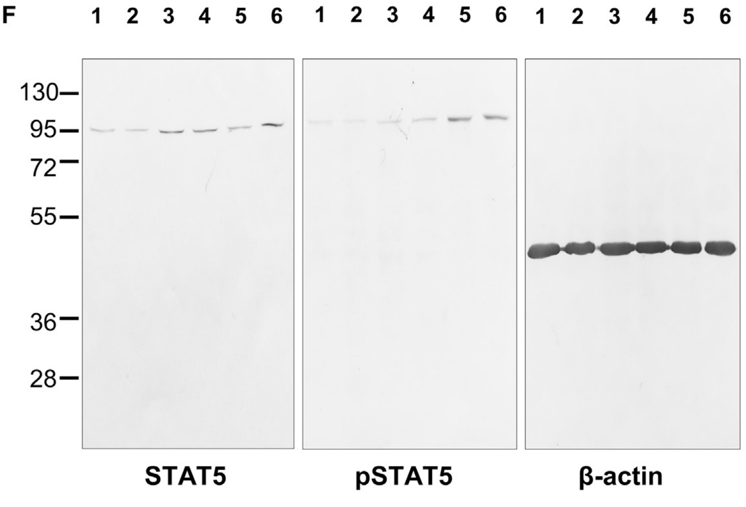

% Signal intensity

	Condition	β-actin	STAT5	pSTAT5
1	w/o	93	79	42
2	w/o	85	86	44
3	TM123 1x10^{-6} M	94	100	52
4	TM123 1x10^{-8} M	88	95	54
5	IL-3	96	91	93
6	GM-CSF	100	94	100

Figure 1. Switchable UniCAR-T Triggered by TM123 Are Highly Active Against AML

(A) Schematic presentation of the inducible modular UniCAR-T platform. Human T cells were genetically engineered to express either functional UniCARs, signaling-deficient UniCARs (UniCAR$_{stop}$), or enhanced green fluorescent protein only (vector control). (B) Upregulation of activation marker CD25 on CD4^{+} and CD4^{-} UniCAR-T (2 × 10^{4}) cultivated with OCI-AML3 cells at an effector to target (e:t) ratio of 1:1 was determined by flow cytometry after 48 h (mean ± SD). (C) Lysis of OCI-AML3 cells after 48 h in the presence of TM123 normalized to controls lacking UniCAR-T (mean ± SD). (D) Detection of IFN-γ in cell culture supernatants after 48 h of cultivation with OCI-AML3 cells (mean ± SEM). (E) TM123 dose-response-dependent cytotoxic efficacy and IFN-γ release of UniCAR-T after 48 h of cultivation with OCI-AML3 cells (mean ± SD). Culture

(legend continued on next page)

evaluated for a series of targets expressed on several hematopoietic[30,31] and solid tumors[32–34] *in vitro* and *in vivo*.[35] Herein, we present data from the preclinical and translational development of a UniCAR-based treatment of acute leukemia. We have demonstrated efficient tumor reactivity *in vitro* and *in vivo* using T cells that were engineered to express a UniCAR construct optimized for clinical applications and redirected against $CD123^+$ leukemia cells.

RESULTS

Redirection of Modular UniCAR-T Using an Optimized CD123-Specific Targeting Module Mediates Efficient *In Vitro* Elimination of CD123-Positive AML

The UniCAR platform technology splits antigen-recognition and receptor signaling properties of CAR-T into two separate operational units.[28] T cells are engineered to express a universal CAR (UniCAR-T) that recognizes a small linear peptide derived from the nuclear human La/SS-B protein (UniCAR epitope [UCE]), which is not presented on the cell surface. Consequently, UniCAR-T remain completely inactive under physiological conditions. Soluble adapters termed targeting modules (TMs), consisting of the UCE linked to an appropriate binding domain, mediate antigen-specific activation of UniCAR-T (Figure 1A). A previously published CD28/CD3ζ UniCAR construct[30] and a CD123-specific TM (TM123) were further optimized for clinical application and pre-clinically explored in the present study. Optimization included replacement or de-immunization of all non-human sequences in the constructs. In order to investigate specific activation of UniCAR-T, gene-engineered cells were cultured with 5 nM TM123 alone or in the presence of antigen-expressing target cells. In addition, UniCAR-T lacking intracellular signaling domains ($UniCAR_{stop}$) or modified with EGFP only (vector control) served as controls. We monitored UniCAR-T activation by CD25 expression. Activation and tumor cell elimination were restricted to UniCAR-T in the presence of both TM123 and $CD123^+$ target cells (Figures 1B, 1C, S1A, and S1B). The CD123-expressing AML cell lines OCI-AML3 and MOLM-13 (Figure S1D) were also found to be significantly lysed in a TM123 concentration-dependent manner (Figures 1E and S1E). Cytokine release was restricted to UniCAR-T cross-linked to target cells via TM123 (Figures 1D and S1C). There was a considerable variation between individual donors in the amount of secreted cytokines by TM123-activated UniCAR-T. An overlay of dose-response curves of cytotoxic activity and cytokine release revealed that concentration of TM123 required for a half-maximal cytotoxic response (EC_{50}; 25 pM) was approximately 10-fold lower than the TM123 concentration that induces a half-maximal cytokine release (Figure 1E). Upon completion of a screen of 34 cytokines, we found interferon γ (IFN-γ), interleukin 2 (IL-2), granulocyte-macrophage colony-stimulating factor (GM-CSF), macrophage-inflammatory protein 1a/b (MIP-1a/b), and IL-1 receptor antagonist (IL-1RA) as the dominant cytokines released by TM123-activated UniCAR-T against OCI-AML3 cells (Figure S2). In a clinical setting, UniCAR-T will be most likely outnumbered by the leukemic blasts, and hence we explored the ability of UniCAR-T to lyse AML cells in a TM123-dependent manner at low effector-to-target cell (e:t) ratios. Cytotoxic responses at different e:t ratios and time points resulted in comparable EC_{50} values (Figure S1F). The expansion of the UniCAR-T population upon TM-mediated cross-linkage was found to be even higher than expansion induced by antibody-mediated CD3/CD28 stimulation (Figure S1G).

An important safety question for clinical application of TM123 was the potential of the TM to induce downstream signaling events upon binding to CD123. Therefore, we investigated the downstream effects of TM123 binding to CD123 via analysis of STAT5 phosphorylation. While STAT5 was detected in all tested conditions, an increase in phosphorylated STAT5 was only found in samples incubated with IL-3 or GM-CSF, but not in samples treated with up to 1 μM TM123 (Figure 1F).

In addition to the AML cell lines, TM123-redirected UniCAR-T were also tested on primary $CD123^+$ AML samples (Figure 2A). The allogeneic UniCAR-T eliminated primary AML blasts at a low e:t ratio of 1:5 after 48 h with an estimated EC_{50} of 22–37 pM, which was comparable to the results obtained with cell lines (Figure 2B).

TM123-Redirected UniCAR-T Efficiently Eliminate CD123-Positive B-ALL Cells *In Vitro*

Beyond AML, CD123 is highly associated with B-lymphoblastic leukemia (B-ALL). Hence, we also investigated the performance of UniCAR-T against TOM-1 cells and primary $CD123^+$ B-ALL samples (Figures 2A and S1H). UniCAR-T redirected to TOM-1 demonstrated potent anti-leukemic effects with an EC_{50} in picomolar concentration of TM123 (Figure S1I). We also found that both allogeneic and autologous UniCAR-T mediated the eradication of primary B-ALL blasts after either 24 or 48 h (Figures 2C and 2D).

TM123 Demonstrates Favorable Pharmacokinetic and Pharmacodynamic Properties for Enabling Rapid Switchability of UniCAR-T

We studied the pharmacokinetics of TM123, as the *in vivo* availability of UniCAR-specific TMs is crucial for their anti-tumor efficacy (Figure 3). Clearance from peripheral blood (PB) after intravenous (i.v.) injection was determined in experimental mice, and a plasma half-life of 27 min was calculated for TM123 (Figure 3A). *In vitro* experiments confirmed that cell-bound TM123 rapidly internalizes upon binding to CD123 at 37°C, with an estimated half-life of 31 min, favoring the rapid switchability of TM123-redirected UniCAR-T (Figure 3B). Furthermore, we found that TM123 infiltrates the bone marrow (BM) immediately after i.v. bolus injection (Figure 3C).

conditions in (C), (D), and (E) are as described for (B). (F) Quantification of phosphorylation of STAT5 upon signaling induction via the GM-CSF/IL-3/IL-5 receptor complex on U937-CD123 cells through binding of TM123 (lanes 3 and 4, respectively), recombinant IL-3 (lane 5), GM-CSF (lane 6), or without exogenous additives (lanes 1 and 2). Statistical significance for (B), (C), and (D) was assessed by nonparametric one-way analysis of variance (ANOVA; Kruskal-Wallis test) with a *post hoc* Dunn's multiple comparison test (*$p \leq 0.05$, **$p \leq 0.01$).

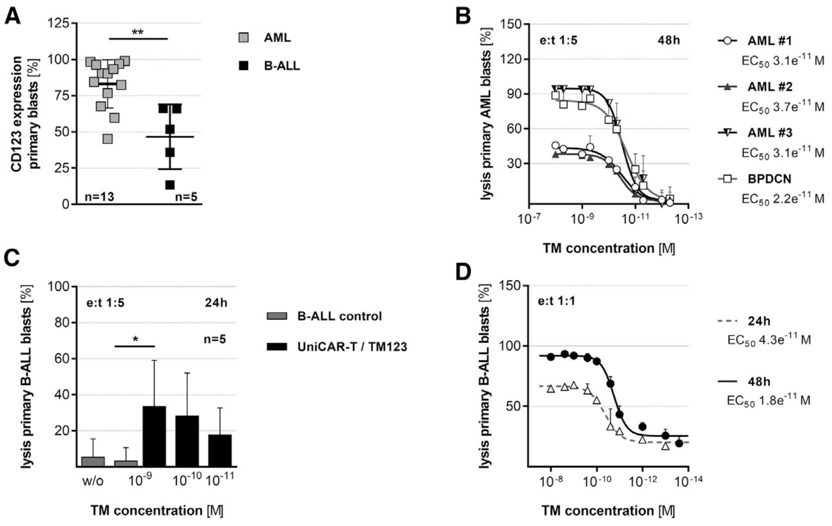

Figure 2. CD19- and CD123-Redirected UniCAR-T Are Highly Efficient at Targeting Primary Acute Leukemia

(A) CD123 expression on patient-derived AML and B-ALL blasts. (B) Lysis of patient-derived primary AML blasts by TM123-redirected allogeneic UniCAR-T at an e:t of 1:5 after 48 h. Results were normalized to allogeneic control samples lacking any TM123 (mean ± SD). (C) TM123-redirected allogeneic UniCAR-T (1×10^4) were cultured with patient-derived B-ALL samples at an e:t of 1:5 and indicated concentrations of TM123. Living leukemic cells were determined after 24 h via flow cytometry. Cytotoxic activity was benchmarked against the tumor cells-only control. (D) TM123-redirected autologous UniCAR-T (2×10^4) were co-cultured with primary B-ALL blasts. EC_{50} concentrations were determined after 24 and 48 h at an e:t of 1:1. Target cell lysis was normalized to control samples without TM (mean ± SD). Statistical significance was assessed by a non-parametric Wilcoxon-Mann-Whitney test (A) or by parametric analysis of variance (ANOVA) with Dunnett's multiple comparison test (C) (*$p \leq 0.05$, **$p \leq 0.01$).

The functionality of administered TM123 was demonstrated by binding of re-isolated TMs from PB and BM to MOLM-13 cells (Figure 3D).

UniCAR-T Demonstrate Anti-Leukemic Efficacy against Acute Leukemia *In Vivo*

In vivo efficacy of TM123-redirected UniCAR-T was explored in a systemic MOLM-13 xenograft model (Figure 4A). Treatment with TM123 via an intraperitoneal (i.p.) injection (Figure S3A) prevented the engraftment of AML and led to a significant survival benefit in tumor-bearing mice (Figure 4B). There was a significant reduction in AML cells in BM and PB within the TM123-treated group (Figure 4C) along with higher $CD3^+$ T cell frequencies measured at each individual endpoint (Figure 4D). The analysis of T cell phenotype[36] was carried out by staining for the surface markers CD3, CD4, CD28, CD45RO, CD95, and CD197. Analysis of the different phenotypes revealed a stable composition of T cell subsets *in vivo* before and after treatment (Figure S3B). Despite control of leukemic progression in the BM during TM123 treatment, the development of mostly subcutaneous metastases at later time points was the predominant cause of death in the TM123-treatment group (data not shown). However, long-term efficacy of TM123-redirected UniCAR-T against MOLM-13 cells was confirmed in an AML Winn-type assay (Figures S4A–S4C). The applied UniCAR-T were isolated 20 weeks after transplantation and re-challenged with MOLM-13 cells *ex vivo*, resulting in a TM123 dose-dependent target cell lysis (Figure S4D). To examine *in vivo* efficacy of UniCAR-T against extramedullary leukemic bulks, we established a patient-derived CD123-expressing B-ALL xenograft (PDX) with locally limited extension. For the extramedullary treatment model (Figures 4E; Figure S3C), blasts were transplanted subcutaneously (s.c.) into the flank of NSG mice. In total, mice received six cycles of TM123 with 2.5 μg/g body weight via i.p. injection for 5 consecutive days. The tumor progression was significantly delayed in UniCAR-T-bearing mice during the application of TM123 (Figures 4F and S3D), leading to a prolonged survival of the treated mice (Figure 4G). However, an increase in tumor mass was observed at the end of therapy. Since UniCAR-T were still detectable at all individual endpoints (Figure S3E), interference of checkpoint molecules could be a possible cause.

Anti-Leukemic Efficacy of TM123-Redirected UniCAR-T is Comparable to CD123 CAR-T

Clinical efficacy data are currently only available for CAR-T harboring a fixed binding moiety. We therefore generated a CAR harboring the TM123 single-chain variable fragment (scFv) as a fixed extracellular binding moiety (Figure 5A). When UniCAR-T and CD123 CAR-T were analyzed through a comparison of their phenotypic subsets,[36] we found that the majority of CAR-T and UniCAR-T exhibited a central memory-like phenotype with a constant $CD4^+$-to-$CD8^+$ ratio (data not shown). Degranulation of UniCAR-T in response to OCI-AML3 cells was restricted to the presence of TM123. Of note, degranulation was observed for both $CD4^+$ and $CD4^-$ UniCAR-T and CD123 CAR-T (Figure 5B). In the case of e:t ratios as low as 1:25, we found effective cytotoxic responses of both CD123 CAR-T and UniCAR-T in the presence of 5 nM TM123. The anti-leukemic *in vitro* efficacy was comparable between TM-redirected UniCAR-T and CD123 CAR-T in all of the conditions analyzed (Figure 5C).

Permanent Antigen Reactivity of CD123 CAR-T is Associated with Hematotoxicity in Humanized Mice

To gain insights into possible effects of antigen density on target cells during an anti-leukemic response of UniCAR-T and CD123 CAR-T, genetically engineered T cells were cultured with $CD123^{high}$- and $CD123^{low}$-expressing target cells (Figure 6A). Both UniCAR-T and CD123 CAR-T efficiently eliminated the $CD123^{high}$ target cells within 120 h. Interestingly, only TM123-redirected UniCAR-T reduced the numbers of $CD123^{low}$ cells to 50% within this time frame,

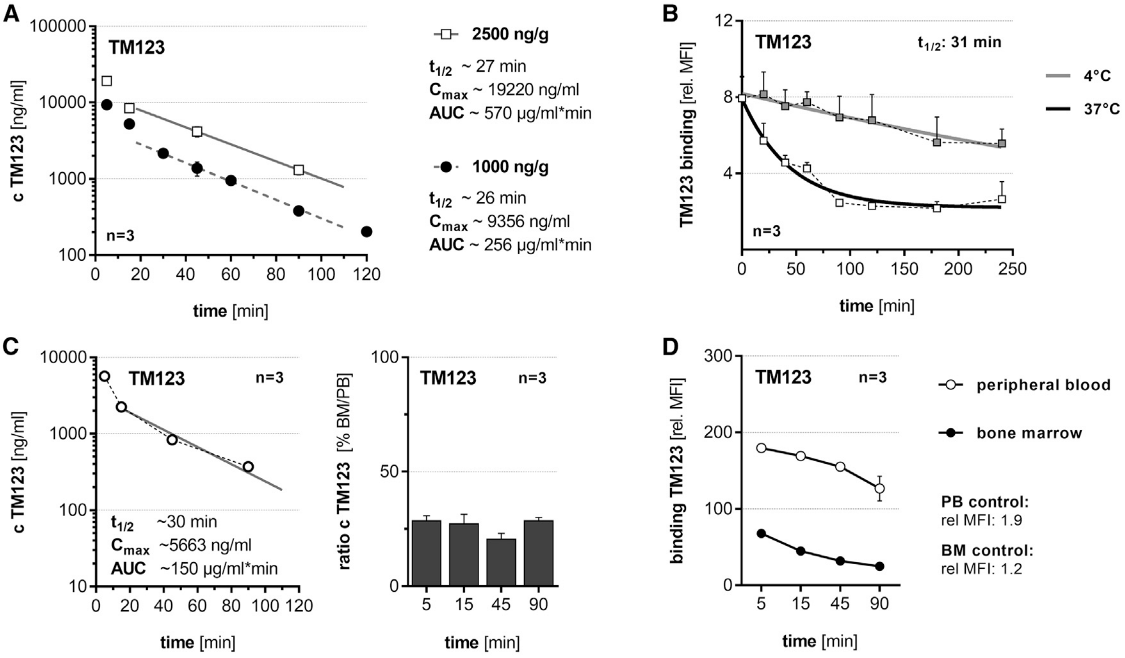

Figure 3. Pharmacokinetics of TM123 Enables Rapid Switching of UniCAR-T

In order to determine half-life of TM123, NSG mice were injected intravenously (i.v.) with indicated amounts of TM123 normalized to the body weight of mice. Samples were taken at the indicated time points. The concentration of TM123 was determined via in-house ELISA.[30] (A) Peripheral blood (PB) pharmacokinetics of 1,000 or 2,500 ng/g TM123 following i.v. bolus. (B) Internalization of TM123 into CD123-positive MOLM-13 cells was measured *in vitro* via flow cytometry at 37°C, while 4°C samples served as a negative control. (C) Bone marrow (BM) infiltration of 2,500 ng/g i.v. injected TM123. The BM infiltration ratio was calculated relative to corresponding plasma samples. (D) Binding of TM123 in PB or corresponding BM samples after i.v. bolus against MOLM-13 cells was determined via flow cytometry. Data represent means of individual experiments or analyzed mice (mean ± SEM).

demonstrating that the modular CAR-T approach is also capable of killing weakly positive cells. After TM123 withdrawal, however, UniCAR-T were switched off and CD123^{low} cells were recovered. In contrast, the continuously active CD123 CAR-T eliminated CD123^{low} cells, albeit with slower kinetics compared to CD123^{high} target cells. These results indicate that temporally and/or concentration-restricted administration of TM123 might allow a differentiated UniCAR-T cytotoxic response against cells with varying CD123 surface densities. We quantified the CD123 surface expression on a large number of cell types using a calibration curve (Figure S5A). Windows for rapid, delayed, and slow cytotoxic responses of UniCAR-T were defined based on results from cytotoxicity assays against cell lines with known CD123 densities (as shown in Figure 6A). CD123 surface density on all analyzed primary AML and B-ALL samples was categorized into the rapid response range with the exception of a sample from a single AML and B-ALL patient each. In contrast, CD34$^+$ hematopoietic stem and progenitor cells (HSPCs) displayed a CD123 surface density triggering a delayed UniCAR-T response (Figure 6B). To examine possible effects of CD123 CAR-T and TM123-redirected UniCAR-T against CD34$^+$ progenitors, we performed cytotoxicity assays. Up to 30–40% lysis of CD34$^+$ progenitors was observed by both TM123-activated UniCAR-T and CD123 CAR-T after 48 h (Figures 6C and S5B). Importantly, no UniCAR-T reactivity was observed in the absence of TM123.

To prove the reversibility of hematotoxicity of TM123-redirected UniCAR-T and to investigate long-term effects of CD123-directed toxicity, NSG mice were injected with pre-incubated cell mixes of allogeneic CD123 CAR-T or UniCAR-T and purified CD34$^+$ HSPCs (Figure 6D). While TM123-redirected UniCAR-T mediated minor toxic effects, the engraftment of human hematopoiesis was not significantly different between mice transplanted with CD34$^+$ HSPCs only and animals co-transplanted with UniCAR-T (Figures 6E, S5C, and S5D). In contrast, continuously active CD123 CAR-T caused significant hematotoxicity, resulting in a longitudinal reduction of human leukocytes in PB (Figures 6E and S5D). Moreover, analysis of BM revealed significantly reduced cell numbers for lineage marker negative cells (Lin$^-$), including the HSC-enriched CD34$^+$ HSPC fraction in mice transplanted with CD123 CAR-T (Figures 6F, S5E, and S5F). We did not detect any significant differences in mice engrafted with UniCAR-T and the control groups.

Good Manufacturing Practice (GMP)-Equivalent Clinical Scale-Manufactured UniCAR-T$_{CS}$ Demonstrate High Efficacy Targeting CD123$^+$ AML Comparable to Laboratory-Scale UniCAR-T

During the establishment and validation of the manufacturing process of clinical-scale UniCAR-T (UniCAR-T$_{CS}$), we performed several runs using leukapheresis products from healthy donors. The

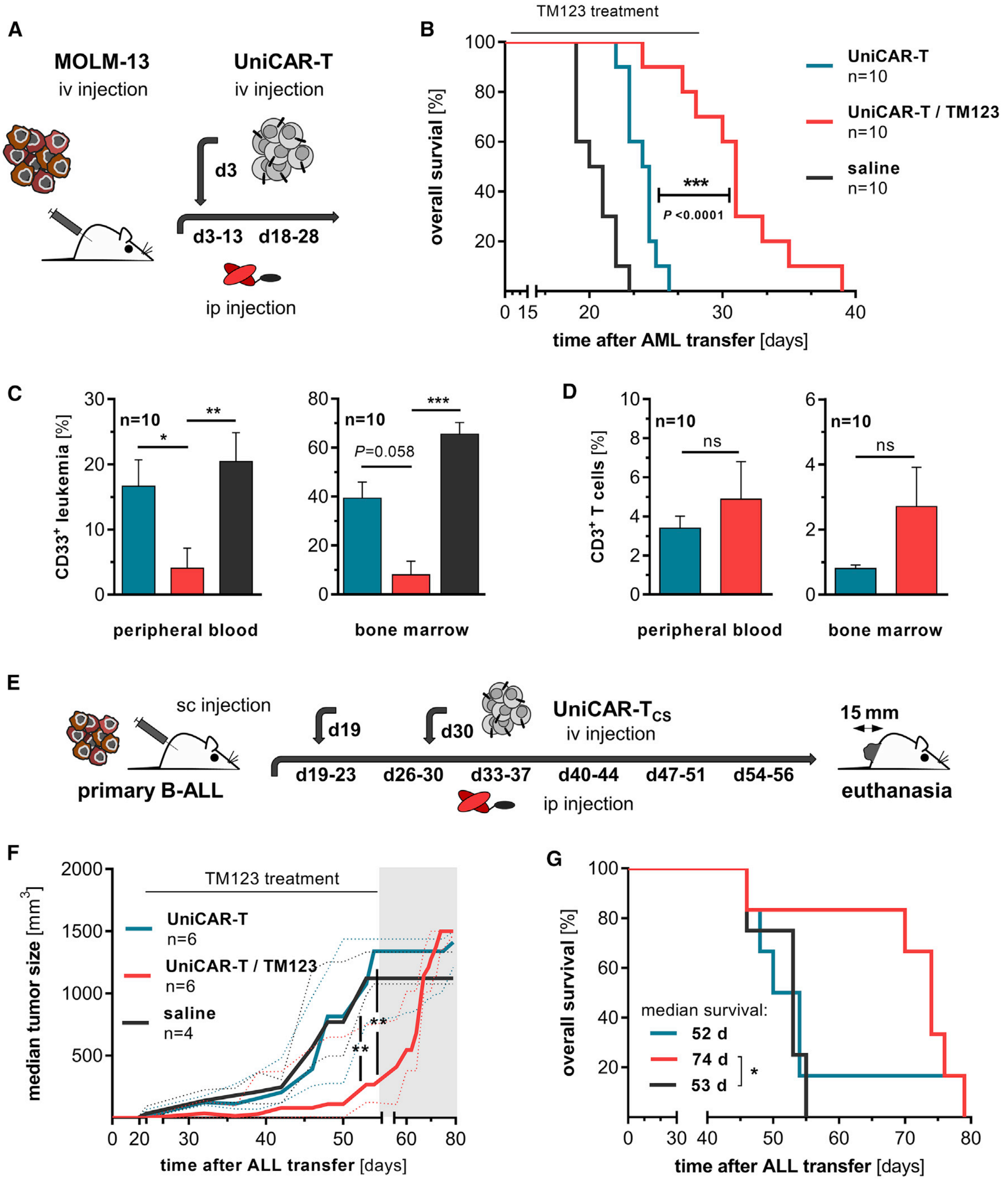

Figure 4. Leukemia Eradication by CD123-Redirected UniCAR-T in NSG CDX and PDX Models

(A) NSG mice were engrafted with 1×10^5 MOLM-13 cells 72 h prior to intravenous (i.v.) transplantation of 5×10^6 UniCAR-T. Mice were injected intraperitoneally (i.p.) with 1 μg/g body weight TM123 twice a day for 10 consecutive days. Treatment was repeated after an application-free period of 5 days. (B) Overall survival of tumor-transplanted mice. (C) Analysis of remaining leukemic cells in the peripheral blood (PB) and bone marrow (BM) of euthanized animals. (D) T cell engraftment of UniCAR-T transplanted mice in PB and BM. A common color code was used for (B), (C), and (D). (E) Patient-derived CD123-positive B-ALL blasts (1×10^6) were transplanted s.c. into the left flank of NSG mice. UniCAR-T were injected i.v. with doses of 2×10^6 and 5×10^6 at 3 (19 days) and 5 (30 days) weeks after tumor application, respectively. Tumor volumes prior to TM123 treatment showed no significant discrepancies among analyzed cohorts. Mice were i.p. injected with 2.5 μg/g body weight TM123 twice a day for 5 consecutive days. Treatment was repeated for a total of six cycles with application-free periods of 2 days in between. The highlighted region indicates the TM123-free period after tumor therapy.

(legend continued on next page)

resulting products had an average frequency of 30% UniCAR-expressing T cells (Figure 7A). Furthermore, UniCAR-T_{CS} were found to predominantly be composed of a memory-like phenotype[36] (central memory [T_{CM}] and transitional memory [T_{TM}] T cells), with minor frequencies of effector memory T cells (T_{EM}) and negligible populations of either stem cell memory (T_{SCM}) or terminal effector (late effector) (T_{LE}) T cells (Figure 7B). The potency and cytokine release of the resulting TM123-redirected cell products were comparable as measured by a standardized cytotoxicity assay (Figures 7C and 7D). Induction of cytokine release occurred at a higher TM123 concentration than specific lysis of target cells and reached half-maximal amounts at TM123 concentrations 3- to 10-fold higher than those required for induction of half-maximal killing (Figures 7C and 7D). Thus, manufacturing at the clinical scale proved to be robust and resulted in rather uniform cell products with anti-leukemic responses comparable to laboratory-scale UniCAR-T batches.

DISCUSSION

There is still a highly unmet medical need for novel therapeutic interventions to treat acute leukemia, in particular for patients with refractory or relapsed disease. Targeted immunotherapy provides a promising option to specifically eliminate chemo-resistant leukemia-initiating cells (LICs), which are reported to be the main cause of relapse.[4] For the treatment of AML and CD19 therapy-resistant B-ALL, CD123 emerged as a suitable target due to its overexpression in AML and B-ALL blasts.[18–21] However, in contrast to CD19, CD123 is a challenging target, as it is also expressed on hematopoietic progenitors[4,22–24] and endothelia.[25,26] Preclinical studies focused on CD123-directed immunotherapy revealed toxicities against normal cells, including $CD34^+$ progenitors.[27–29,37,38] Early clinical trials with CD123-directed CAR-T have not reported major toxicities so far,[39] but CAR-T are currently explored as bridging therapy to allo-HSCT to mitigate potential long-term hematotoxic effects (e.g., ClinicalTrials.gov: NCT02159495). To overcome this limitation, we evaluated the therapeutic potential of our rapidly switchable universal CAR-T platform (UniCAR)[40] for its ability to target CD123-expressing leukemia. We hypothesize that a controlled activation of CAR-T would not only maintain full anti-leukemic efficacy, but also increase safety with no need for safety switch mechanisms via suicide genes or mAb-mediated T cell depletion.[41]

We recently described a bi-specific TM that simultaneously targets CD123 and CD33 and that showed promising results *in vitro* and *in vivo* for anti-tumor efficacy.[30] However, for several reasons we chose a CD123-only TM for further clinical development. First, both CD123 and CD33 are present in major subsets of HSPCs and the myeloid compartment, raising safety concerns about the clinical application of a TM that simultaneously attacks both antigens. Second, bi-specific T cell engagers targeting CD33 are already in clinical studies (ClinicalTrials.gov: NCT03516760 and NCT02520427). Lastly, the development of a CD123-specific TM can also be used for treatment of B-ALL. Hence, we developed a soluble small-sized CD123-specific TM (TM123) that can easily penetrate the BM, the primary site for the localization of residual LICs in AML and B-ALL. In AML, LICs act as key players in relapse after initial treatment[42–46] due to their high self-renewal capacities and chemoresistance.[47,48] As the AML-inducing compartment is described by a $CD34^+/CD38^{-/+}/CD123^+$ immunophenotype,[4,45,49] targeting CD123 potentially enables sustained responses in patients due to eradication of LICs. In the case of B-ALL, LICs[50] also highly express CD123 in the absence of CD19. Thus, combinatorial targeting of CD19 and CD123 could potentially increase the therapeutic response.[20]

We show that UniCAR-T are highly effective, eliminating CD123-positive leukemic cell lines and primary blasts in combination with TM123. In the presence of CD123-expressing target cells, activation and induction of effector functions of UniCAR-T are strictly dependent on the availability of TM123. Conversely, TM123 does not activate UniCAR-T in the absence of CD123-expressing target cells. Moreover, TM123-redirected UniCAR-T were shown to be highly efficient in treating AML and B-ALL in xenograft models by significantly prolonging the survival of mice. The combined UniCAR-T/TM123 treatment also delayed the progression of established extramedullary bulky disease in a B-ALL PDX model.

UniCAR-T efficiently lysed primary AML and B-ALL at picomolar concentrations of TM123 with comparable anti-leukemic efficacy as classical CD123-specific CAR-T. Surprisingly, we observed the half-maximal cytolytic activity of TM123-redirected UniCAR-T at TM concentrations that only induced negligible cytokine release. Thus, there might be a therapeutic window to induce effective killing of leukemic cells by UniCAR-T at TM123 concentrations too low to induce massive cytokine release. Fine-tuning UniCAR-T reactivity with a balanced administration of TM123 may reduce acute adverse events and would even allow the treatment of patients with bulky leukemic burdens. In line with other preclinical studies,[27] CD123 CAR-T significantly impaired human hematopoiesis in our preclinical model. However, only limited cytotoxic response against CD123-expressing HSPCs upon short-term *in vitro* cultivation with CD123 CAR engrafted cytokine-induced killer (CIK) cells was reported by others,[28,51,52] comparable to results for short-term TM123-activated UniCAR-T during this study. The *in vitro* treatment of HSPCs with TM123-redirected UniCAR-T resulted in lysis of presumably CD123-expressing HSPCs to a similar extent as observed for

(F) Progression of extramedullary bulky disease was monitored during an observation period of 12 weeks utilizing a digital caliper. Data represent longitudinal median tumor growth of treated cohorts (median ± range). For analysis of tumor progression of all individual mice, please refer to Figure S3D. (G) Kaplan-Meier survival analysis in the experimental groups. Statistical significance for (B) and (G) was assessed by the Kaplan-Meier method with log rank (Mantel-Cox), non-parametric one-way analysis of variance (Kruskal-Wallis test) with Dunn's multiple comparison test (C), a non-parametric Wilcoxon-Mann-Whitney test (D), or a two-way analysis of variance (ANOVA) (F) (*p $\leq$ 0.05, **p $\leq$ 0.01, ***p $\leq$ 0.001; ns, not significant).

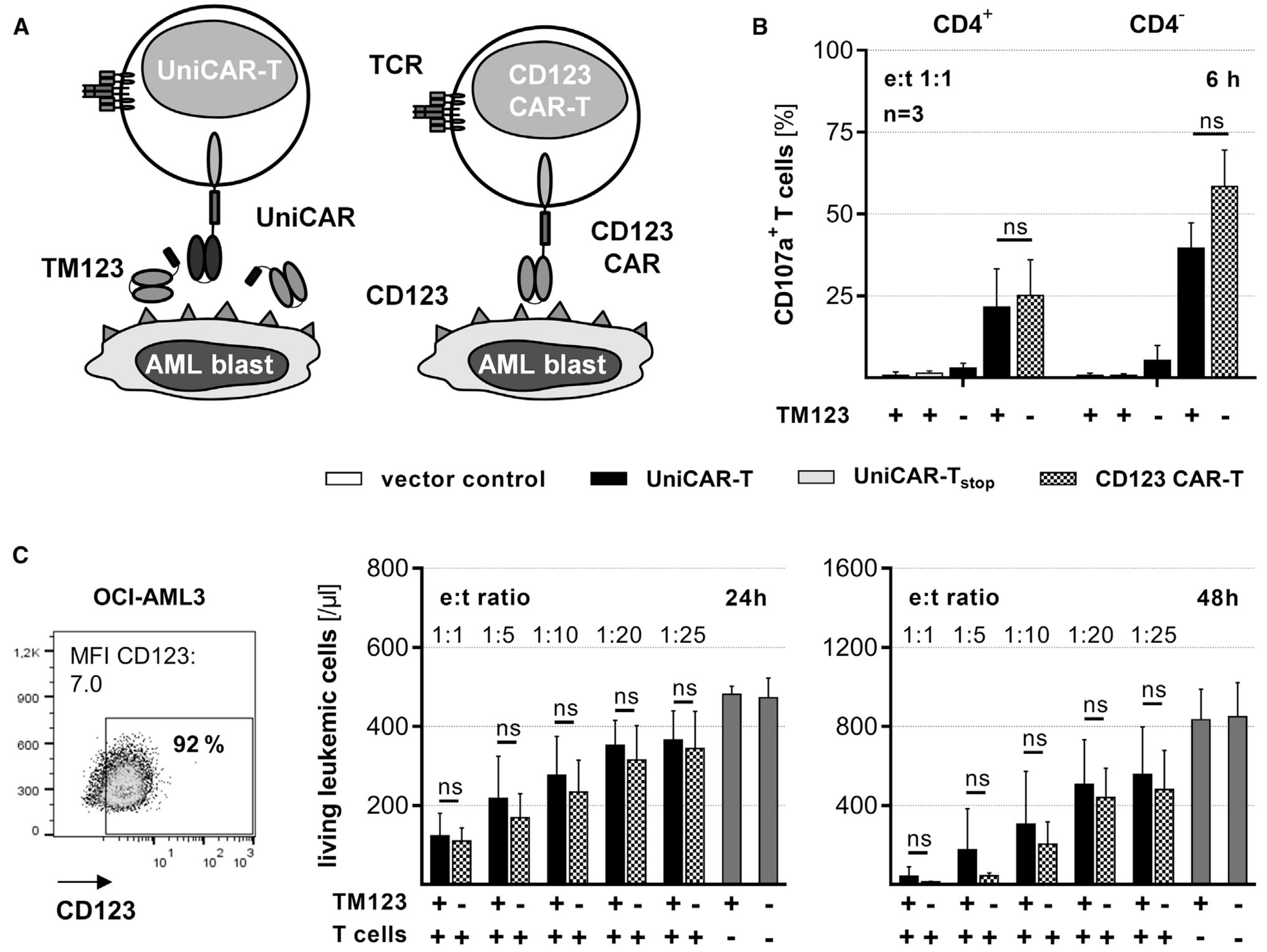

Figure 5. Anti-Leukemic Efficacy of TM123-Redirected UniCAR-T Is Comparable to CD123 CAR-T

(A) A CD123-specific CAR construct including a fixed binding moiety was constructed by replacing the UniCAR binding domain with the single-chain fragment variable (scFv) of the CD123-specific TM. (B) CD123 CAR-T as well as UniCAR-T (2×10^4) in the presence or absence of 5 nM TM123 were cultivated with OCI-AML3 cells at an e:t ratio of 1:1. Degranulation of CAR-T and UniCAR-T was determined by CD107a expression after 6 h of culture. (C) Cytotoxic efficacy of CD123 CAR-T (n = 3) and UniCAR-T (n = 5) in combination with 5 nM TM123 against OCI-AML3 cells (5×10^4) was determined after 24 and 48 h for the indicated range of e:t ratios and compared to control samples containing tumor cells only (gray bars). Statistical significance was assessed for indicated numbers of donors by non-parametric one-way analysis of variance (Kruskal-Wallis test) with Dunn's multiple comparison test (B) or a non-parametric Wilcoxon-Mann-Whitney test (C) (*$p \leq 0.05$; ns, not significant).

CD123 CAR-T. However, this effect is transient and abrogated by TM123 withdrawal, since no significant impact was observed *in vivo*. In this line, UniCAR-T alone did not exhibit any toxicity against HSPCs.

Other modular CAR-T approaches made use of larger antibody fragments redirecting CAR-T against targets with better safety profiles, e.g., CD19 or CD20 (ClinicalTrials.gov: NCT02776813).[53–55] However, we think that the pharmacokinetic properties of full-length antibodies such as rituximab are not suitable for the control of a fast-acting CAR-T system. The short plasma half-life of TM123 (<1 h) with an internalization rate of less than 2 h leads to its rapid elimination and provides a fast mechanism for the systemic switch-off of UniCAR-T activity within less than 4 h. Notably, the safety of flotetuzumab (MGD006), a CD3×CD123 bi-specific antibody with a short plasma half-life that is comparable to that of TM123, was pre-clinically explored in non-human primate models,[56] wherein repeated treatments even with high doses resulted in a nonsignificant reduction of the $CD34^+/CD38^+$ HSPCs, and no effect on endothelia and $CD34^+/CD38^-$ cells was reported.[56] Moreover, flotetuzumab was reported to have an acceptable safety profile during clinical investigation.[57,58]

Overall, the preclinical data accumulated in the present study provide a strong foundation for supporting the exploration of safety and efficacy of the TM123-redirected UniCAR-T in a phase I clinical trial (ClinicalTrials.gov: NCT04230265). The modular approach of the

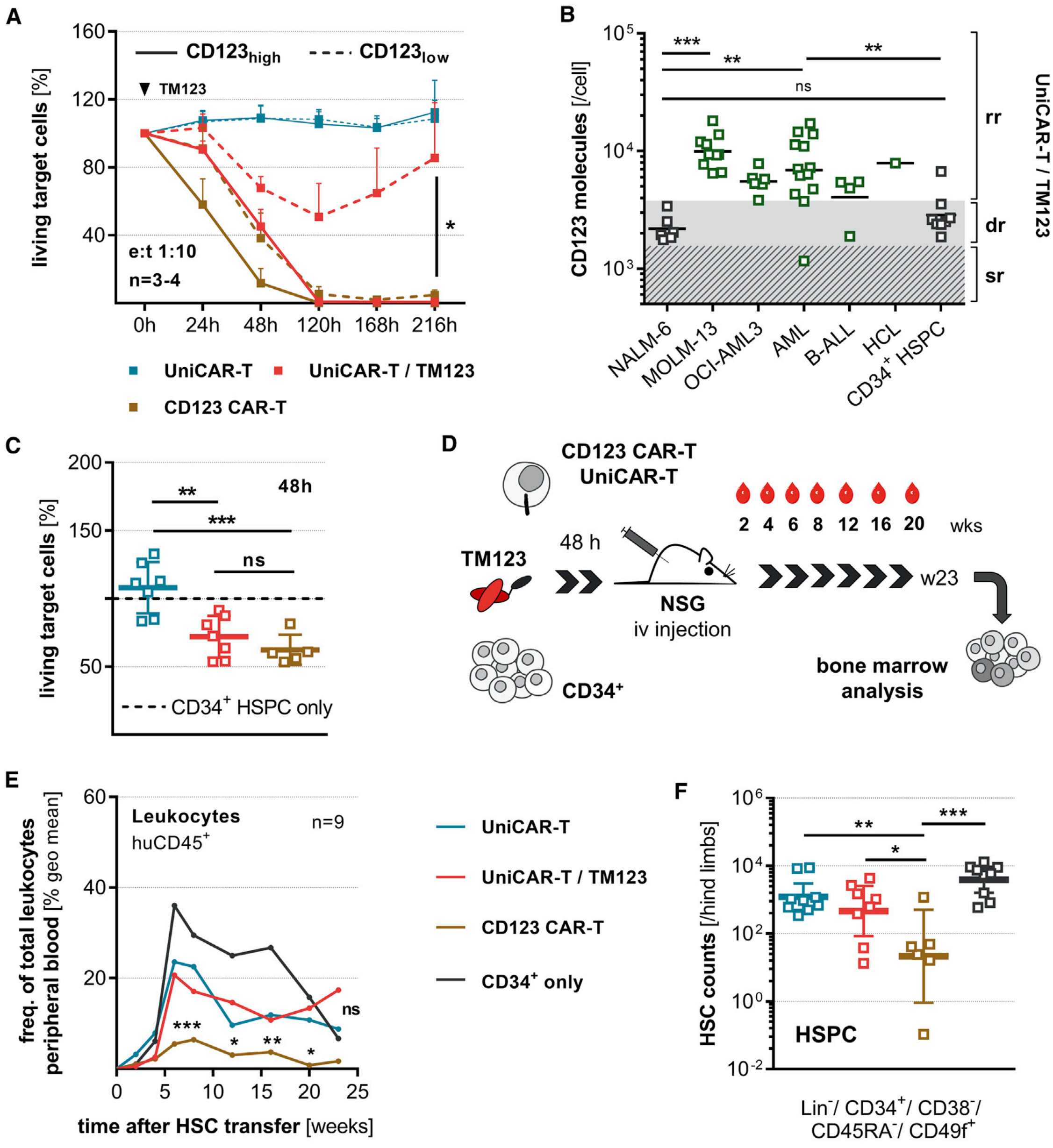

Figure 6. Hematotoxic Effects of Constitutively Active CD123 CAR-T in Humanized Mice Are Prevented By the Switch-Off Mechanism of UniCAR-T

(A) UniCAR-T or CD123 CAR-T (2×10^3) were incubated with 1:1 pre-mixed CD123^{high}- and CD123^{low}-expressing cells (OCI-AML3, NALM-6) at an e:t ratio of 1:10. TM123 was added once at the start of the experiment in the respective groups. Long-term cytotoxic responses against individual target cell populations were monitored for 216 h (mean ± SEM). (B) CD123 was quantified via an antigen standard curve on cell lines, patient-derived leukemia samples, and CD34-purified hematopoietic stem and progenitor cells (HSPCs) from granulocyte colony-stimulating factor (G-CSF)-mobilized healthy donors. Rapid (rr), delayed (dr), and slow response (sr) ranges of UniCAR-T with TM123 have been adapted to pre-determined anti-tumor efficacy rates against several CD123-expressing cell lines. (C) CD34$^+$ HSPCs purified from G-CSF-mobilized healthy donors were co-cultured with allogeneic CD123 CAR-T or UniCAR-T with or without 5 nM TM123 at an e:t ratio of 1:2. Cytotoxic response against hematopoietic progenitors was determined after 48 h of incubation of CD34$^+$ HSPCs (1×10^5) with CAR-T (5×10^4). Results were normalized to control samples containing CD34$^+$ progenitors only. (D) Mixtures of CD34$^+$ HSPCs (1×10^6) and CD123 CAR-T or UniCAR-T (5×10^5) combined with or without 5 nM TM123 cultured for 48 h were subsequently injected into sub-lethally irradiated NSG mice. Peripheral blood (PB) samples were taken up to 20 weeks after transplantation. (E) Engraftment analysis of CD45$^+$ human leukocytes within PB. Significance levels were calculated relative to the control group injected with CD34$^+$ cells only. (F) Bone marrow (BM) samples were analyzed for the presence of the lineage marker negative compartment (Lin$^-$) and in particular for the hematopoietic stem cell-enriched cell pool via flow cytometry 23 weeks after transplantation (geometric mean ± 95% confidence interval [CI]). For analysis of human cell engraftment for all individual mice, see also Figure S5D. Statistical significance was assessed by a parametric one-way analysis of variance (ANOVA) with Tukey's (A and B) or Dunnett's multiple comparison test (C and F) and nonparametric ANOVA (Kruskal-Wallis test) with a *post hoc* Dunn's multiple comparison test (E) (*$p \leq 0.05$, **$p \leq 0.01$, ***$p \leq 0.001$).

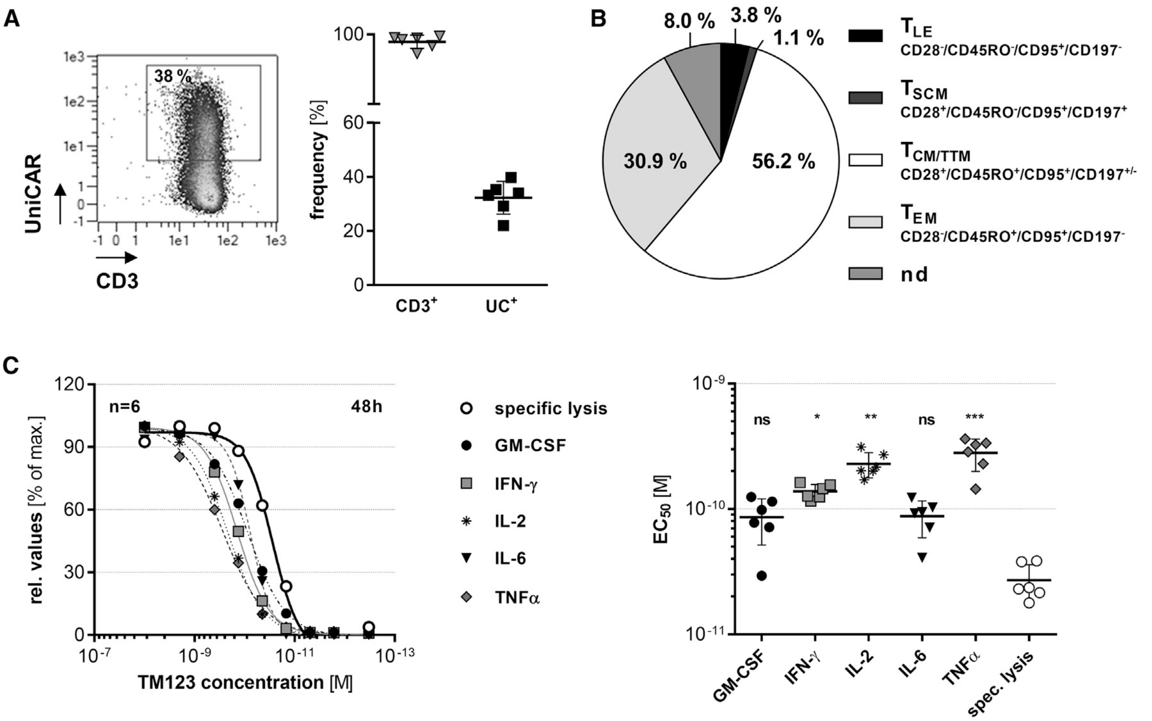

D

cTox	Donor I	Donor II	Donor III	Donor IV	Donor V	Donor VI
EC_{50} [M]	3.9×10^{-11}	2.3×10^{-11}	1.8×10^{-11}	2.2×10^{-11}	2.4×10^{-11}	3.8×10^{-11}

n=6	cTox	GM-CSF	IFN-γ	IL-2	IL-6	TNFα
EC_{50} [M]	2.8×10^{-11}	8.6×10^{-11}	1.4×10^{-10}	2.3×10^{-10}	8.0×10^{-11}	2.8×10^{-10}
Fold difference	1	3.2	5.1	8.4	3.0	10.3

Figure 7. Clinical Scale-Manufactured UniCAR-T_{CS} Demonstrate High Efficacy Targeting $CD123^+$ AML

Good manufacturing practice (GMP)-equivalent manufactured clinical-scale UniCAR-T_{CS} were generated utilizing the CliniMACS Prodigy® automated system. (A) UniCAR surface expression of clinical-scale runs was determined via flow cytometry staining with a monoclonal antibody specific for the extracellular linker domain of the UniCAR. (B) UniCAR-T_{CS} phenotype[36] in the final cell product from six representative batches was estimated via staining for the surface markers CD3, CD4, CD28, CD45RO, CD95, and CD197. UniCAR-T were classified in late effector (T_{LE}), stem cell memory (T_{SCM}), central memory/transitional memory (T_{CM}/T_{TM}), and effector memory (T_{EM}) T cells.[36] Non-defined cells (nd) fall outside of these definitions. (C) TM123 dose-response-dependent cytotoxic efficacy and cytokine release of UniCAR-T_{CS} after 48 h of cultivation with OCI-AML3 cells. Half-maximal response (EC_{50}) for cytokine secretion and specific lysis were calculated from response curves (mean ± SD). Data were further normalized against the maximum value observed in each dataset. (D) Donor-specific data for plots shown in (C). Statistical significance was assessed by non-parametric one-way analysis of variance (Kruskal-Wallis test) with a *post hoc* Dunn's multiple comparison test (*$p \leq 0.05$, **$p \leq 0.01$, ***$p \leq 0.001$; ns, not significant).

UniCAR platform technology adds rapid control mechanisms for CAR-T reactivity to allow targeting of antigens with potential safety concerns, while maintaining the high anti-leukemic activity of CAR-T. Moreover, a combination of, or sequential application of, several TMs during treatment might prevent development of therapy-induced antigen-loss escape variants in the future.

MATERIALS AND METHODS

Cell Culture

TOM-1 (DMSZ, ACC 578) and MOLM-13 cells (DMSZ, ACC 554) were cultured in complete RPMI 1640 medium supplemented with 20% or 10% fetal calf serum (FCS), respectively (all purchased from Merck Millipore, Burlington, MA, USA). HEK293T (ATCC, CRL-11268), HT-1080 (ATCC, CCL-121), and OCI-AML3 cells (DMSZ, ACC 582) were cultured in DMEM, high glucose, GlutaMAX (Thermo Fisher Scientific, Waltham, MA, USA) supplemented with 10% FCS. Primary AML blasts and $CD34^+$ HSPCs were cultured in stem cell growth medium (SCGM) (CellGenix, Portsmouth, NH, USA) supplemented with 1% human serum albumin (HSA) (Baxter, Deerfield, IL, USA), 10 ng/mL Flt3L, 10 ng/mL SCF, and 10 pg/mL IL-3 (all purchased from PeproTech, Rocky Hill, NJ, USA). ALL blasts were cultured in adapted Iscove's modified Dulbecco's medium

(IMDM) medium[59] (Thermo Fisher Scientific, Waltham, MA, USA). Mycoplasma tested cells were maintained at 37°C in a humidified atmosphere of 5% CO_2.

Design of CAR Constructs and Manufacturing of CAR-T Batches

The design of the UniCAR harboring CD28/CD3ζ-derived signaling domains was already described.[30,60] For the clinical construct, structural modifications were made in order to reduce immunogenicity and enhance the UniCAR framework design. To generate CAR-T batches, human T cells were isolated from leukapheresis product of healthy donors (Cellex, Dresden, Germany) or purified from buffy coats (German Red Cross, Dresden, Germany).[30] CAR expression on T cells was analyzed as described recently.[30,61] GMP-equivalent manufactured UniCAR-T_{CS} were generated utilizing the CliniMACS Prodigy (Miltenyi Biotec, Bergisch Gladbach, Germany) automated system and clinical-grade retroviral supernatant.

Flow Cytometry

Experiments were performed on a MACSQuant Analyzer 10 (Miltenyi Biotec, Bergisch Gladbach, Germany) or BD LSR II (BD Biosciences, Franklin Lakes, NJ, USA) and analyzed via FlowJo v10 software (FlowJo, Ashland, OR, USA). Antibodies used for analysis of cells were as follows: anti-CD4 (SK3), anti-CD8 (RPA-T8), anti-CD10 (HI10a), anti-CD14 (M5E2), anti-CD19 (HIB-19), anti-CD25 (BC96), anti-CD28 (CD28.2), anti-CD33 (WM53), anti-CD34 (8G12), anti-CD38 (HIT2), anti-CD45RO (UCHL1), anti-CD45RA (HI100), anti-CD66b (G10F5), anti-CD95 (DX2), anti-CD107a (H4A3), anti-CD123 (9F5), and anti-CD197 (150503) (all purchased from BD Biosciences, Franklin Lakes, NJ, USA); anti-human leukocyte antigen (HLA)-DR (L243) and anti-CD3 (UCHT1) (both purchased from Thermo Fisher Scientific, Waltham, MA, USA); anti-CD45 (HI30), anti-CD123 (6H6), and anti-HuLin (OKT3/M5E2/3G8/HIB19/2H7/HCD56) (all purchased from BioLegend, San Diego, CA, USA); anti-CD49f (REA518) (Miltenyi Biotec, Bergisch Gladbach, Germany); and anti-La (5B9) and anti-La (7B6)[30,61] (in-house) or GaM-Alexa Fluor 647 (Dianova, Hamburg, Germany). Antigen quantification was performed using Dako QIFIKIT (Agilent Technologies, Santa Clara, CA, USA).

Design, Expression, and Purification of the Recombinant TM TM123

Compared to the CD123-specific TM that has already been published,[30] we optimized TM123 further to mitigate potential immunogenicity of non-human sequences. The CD123-directed scFv derived from a murine mAb was modified by de-immunization. Recombinant TM was expressed in Chinese hamster ovarian (CHO) cells and purified as recently described.[30] Batches were characterized by UV absorption, SDS-PAGE, immunoblot, and high-performance liquid chromatography (HPLC) for content, purity, and identity.

Determination of T Cell Activation and Cytotoxic Responses

T cell activation and cytotoxic responses were determined via flow cytometry as described before.[30] Tumor elimination was normalized to donor-specific allogeneic controls. Primary leukemic blasts were identified via flow cytometry. Expansion of CAR-T was ratio-calculated to seeded numbers of cells. T cell degranulation was analyzed via staining of CD107a after 6 h. Accordingly, monensin (BioLegend, San Diego, CA, USA) was added to cytotoxicity assays containing anti-CD107a mAb after 1 h of incubation.

Determination of Cytokine Release

Cell-free supernatants were analyzed by BD OptEIA sandwich ELISA kits (BD Biosciences, Franklin Lakes, NJ, USA) or Cytokine & Chemokine 34-Plex Human ProcartaPlex Panel 1A (Thermo Fisher Scientific, Waltham, MA, USA) according to the manufacturer's protocols.

Quantification of Phosphorylation of STAT5 upon Signaling Induction via the GM-CSF/IL-3/IL-5 Receptor Complex

U937-CD123 cells were incubated with 1×10^{-6} or 1×10^{-8} M TM123 or recombinant IL-3 (5 ng/mL) or GM-CSF (15 ng/mL) (PeproTech, Rocky Hill, NJ, USA) or without exogenous additives for 15 or 30 min.[62] Cell lysates were prepared and gels loaded with equal volumes for immunoblotting. STAT5, phosphorylated STAT5 (pSTAT5), and β-actin (loading control) were detected using anti-STAT5 (D3N2B), anti-pSTAT5 (Tyr694) (D47E7), anti-β-actin (D6A8) and an alkaline phosphatase-linked anti-rabbit immunoglobulin G (IgG) (all purchased from Cell Signaling Technology, Danvers, MA, USA). Protein content was quantified using Image Lab software (v5.2.1, build 11). Relative signal intensities were referenced to the GM-CSF-treated sample.

NSG Xenograft Models

Animal experiments were performed with 8- to 12-week-old male and female NSG mice according to the German animal protection law (LDS, TVV 61/2017, TVV 87/2017). Leukemic cells were either applied i.v. via tail vein injection or injected s.c., whereas UniCAR-T always were transplanted i.v. The s.c. transplanted grafts were premixed in Matrigel (Corning Life Sciences, Corning NY, USA) (1:1) and injected into the flank. Engraftment of human $CD34^+$ cells was enhanced by sublethal total-body irradiation 24 h prior transplantation. Sample preparation was performed as described elsewhere.[30]

Statistical Analysis

Tests indicated in figure legends were performed using GraphPad Prism 6 (GraphPad, La Jolla, CA, USA). p values <0.05 were considered significant.

SUPPLEMENTAL INFORMATION

Supplemental Information can be found online at https://doi.org/10.1016/j.omto.2020.04.009.

AUTHOR CONTRIBUTIONS

S.L., A.E., M.C., and J.R. designed experiments; S.L., J.D., J.-E.M., and J.R. performed experiments; S.L., J.-E.M., and J.R. analyzed data; M.v.B., J.S., J.R., A.F., M.B., and G.E. provided materials; S.L., A.E., and M.C. wrote the manuscript; S.L., J.D., J.S., J.R., M.v.B., M.S., C.G., K.F., A.E., and M.C. reviewed the manuscript.

CONFLICTS OF INTEREST

M.B., G.E., and A.E. are shareholders of GEMoaB Monoclonals GmbH. G.E. and A.E. are shareholders of Cellex Patient Treatment GmbH. S.L., J.D., J.-E.M., J.S., J.R., C.G., M.S., K.F., A.E., and M.C. are employees at GEMoaB Monoclonals GmbH or Cellex Patient Treatment GmbH. M.v.B. is a consultant to both companies.

ACKNOWLEDGMENTS

We thank Glenn Gröbe, Juliane Schneider, Christin Eger, Susann Gerber, Sarah Tröger, Nicole Kronstein, Sonja Schallenberg, Kathrin Zimmermann, Ilka Müller, and Robert Kuhnert for assistance. This work was supported by GEMoaB Monoclonals GmbH, Cellex Patient Treatment GmbH, and grants from the German Federal Ministry of Education and Research (BMBF; FKZ01EK1513B), the European Social Fund, and the Free State of Saxony.

REFERENCES

1. Ferrara, J.L., and Reddy, P. (2006). Pathophysiology of graft-versus-host disease. Semin. Hematol. *43*, 3–10.
2. Dykewicz, C.A.; Centers for Disease Control and Prevention (U.S.); Infectious Diseases Society of America; American Society of Blood and Marrow Transplantation (2001). Summary of the Guidelines for Preventing Opportunistic Infections among Hematopoietic Stem Cell Transplant Recipients. Clin. Infect. Dis. *33*, 139–144.
3. Coiffier, B., Lepage, E., Brière, J., Herbrecht, R., Tilly, H., Bouabdallah, R., Morel, P., Van Den Neste, E., Salles, G., Gaulard, P., et al. (2002). CHOP chemotherapy plus rituximab compared with CHOP alone in elderly patients with diffuse large-B-cell lymphoma. N. Engl. J. Med. *346*, 235–242.
4. Jin, L., Lee, E.M., Ramshaw, H.S., Busfield, S.J., Peoppl, A.G., Wilkinson, L., Guthridge, M.A., Thomas, D., Barry, E.F., Boyd, A., et al. (2009). Monoclonal antibody-mediated targeting of CD123, IL-3 receptor α chain, eliminates human acute myeloid leukemic stem cells. Cell Stem Cell *5*, 31–42.
5. Kantarjian, H., Stein, A., Gökbuget, N., Fielding, A.K., Schuh, A.C., Ribera, J.M., Wei, A., Dombret, H., Foà, R., Bassan, R., et al. (2017). Blinatumomab versus chemotherapy for advanced acute lymphoblastic leukemia. N. Engl. J. Med. *376*, 836–847.
6. Neelapu, S.S., Locke, F.L., Bartlett, N.L., Lekakis, L.J., Miklos, D.B., Jacobson, C.A., Braunschweig, I., Oluwole, O.O., Siddiqi, T., Lin, Y., et al. (2017). Axicabtagene ciloleucel CAR T-cell therapy in refractory large B-cell lymphoma. N. Engl. J. Med. *377*, 2531–2544.
7. Park, J.H., Rivière, I., Gonen, M., Wang, X., Sénéchal, B., Curran, K.J., Sauter, C., Wang, Y., Santomasso, B., Mead, E., et al. (2018). Long-term follow-up of CD19 CAR therapy in acute lymphoblastic leukemia. N. Engl. J. Med. *378*, 449–459.
8. Schuster, S.J., Svoboda, J., Chong, E.A., Nasta, S.D., Mato, A.R., Anak, Ö., Brogdon, J.L., Pruteanu-Malinici, I., Bhoj, V., Landsburg, D., et al. (2017). Chimeric antigen receptor T cells in refractory B-cell lymphomas. N. Engl. J. Med. *377*, 2545–2554.
9. Gardner, R.A., Finney, O., Annesley, C., Brakke, H., Summers, C., Leger, K., Bleakley, M., Brown, C., Mgebroff, S., Kelly-Spratt, K.S., et al. (2017). Intent-to-treat leukemia remission by CD19 CAR T cells of defined formulation and dose in children and young adults. Blood *129*, 3322–3331.
10. Maude, S.L., Frey, N., Shaw, P.A., Aplenc, R., Barrett, D.M., Bunin, N.J., Chew, A., Gonzalez, V.E., Zheng, Z., Lacey, S.F., et al. (2014). Chimeric antigen receptor T cells for sustained remissions in leukemia. N. Engl. J. Med. *371*, 1507–1517.
11. Bach, P.B., Giralt, S.A., and Saltz, L.B. (2017). FDA Approval of tisagenlecleucel: promise and complexities of a $475 000 cancer drug. JAMA *318*, 1861–1862.
12. Turtle, C.J., Hanafi, L.A., Berger, C., Gooley, T.A., Cherian, S., Hudecek, M., Sommermeyer, D., Melville, K., Pender, B., Budiarto, T.M., et al. (2016). CD19 CAR-T cells of defined $CD4^{+:}CD8^{+}$ composition in adult B cell ALL patients. J. Clin. Invest. *126*, 2123–2138.
13. Locke, F.L., Neelapu, S.S., Bartlett, N.L., Siddiqi, T., Chavez, J.C., Hosing, C.M., Ghobadi, A., Budde, L.E., Bot, A., Rossi, J.M., et al. (2017). Phase 1 results of ZUMA-1: a multicenter study of KTE-C19 anti-CD19 CAR T cell therapy in refractory aggressive lymphoma. Mol. Ther. *25*, 285–295.
14. Grupp, S.A., Laetsch, T.W., Buechner, J., Bittencourt, H., Maude, S.L., Verneris, M.R., Myers, G.D., Boyer, M.W., Rives, S., and De Moerloose, B. (2016). Analysis of a global registration trial of the efficacy and safety of CTL019 in pediatric and young adults with relapsed/refractory acute lymphoblastic leukemia (ALL). Blood *128*, 221.
15. DeFrancesco, L. (2017). CAR-T's forge ahead, despite Juno deaths. Nat. Biotechnol. *35*, 6–7.
16. Venkataraman, G., Aguhar, C., Kreitman, R.J., Yuan, C.M., and Stetler-Stevenson, M. (2011). Characteristic CD103 and CD123 expression pattern defines hairy cell leukemia: usefulness of CD123 and CD103 in the diagnosis of mature B-cell lymphoproliferative disorders. Am. J. Clin. Pathol. *136*, 625–630.
17. Fromm, J.R. (2011). Flow cytometric analysis of CD123 is useful for immunophenotyping classical Hodgkin lymphoma. Cytometry B Clin. Cytom. *80*, 91–99.
18. Chaperot, L., Bendriss, N., Manches, O., Gressin, R., Maynadie, M., Trimoreau, F., Orfeuvre, H., Corront, B., Feuillard, J., Sotto, J.J., et al. (2001). Identification of a leukemic counterpart of the plasmacytoid dendritic cells. Blood *97*, 3210–3217.
19. Del Giudice, I., Matutes, E., Morilla, R., Morilla, A., Owusu-Ankomah, K., Rafiq, F., A'Hern, R., Delgado, J., Bazerbashi, M.B., and Catovsky, D. (2004). The diagnostic value of CD123 in B-cell disorders with hairy or villous lymphocytes. Haematologica *89*, 303–308.
20. Ruella, M., Barrett, D.M., Kenderian, S.S., Shestova, O., Hofmann, T.J., Perazzelli, J., Klichinsky, M., Aikawa, V., Nazimuddin, F., Kozlowski, M., et al. (2016). Dual CD19 and CD123 targeting prevents antigen-loss relapses after CD19-directed immunotherapies. J. Clin. Invest. *126*, 3814–3826.
21. Ehninger, A., Kramer, M., Röllig, C., Thiede, C., Bornhäuser, M., von Bonin, M., Wermke, M., Feldmann, A., Bachmann, M., Ehninger, G., and Oelschlägel, U. (2014). Distribution and levels of cell surface expression of CD33 and CD123 in acute myeloid leukemia. Blood Cancer J. *4*, e218.
22. Taussig, D.C., Pearce, D.J., Simpson, C., Rohatiner, A.Z., Lister, T.A., Kelly, G., Luongo, J.L., Danet-Desnoyers, G.A., and Bonnet, D. (2005). Hematopoietic stem cells express multiple myeloid markers: implications for the origin and targeted therapy of acute myeloid leukemia. Blood *106*, 4086–4092.
23. Sato, N., Caux, C., Kitamura, T., Watanabe, Y., Arai, K., Banchereau, J., and Miyajima, A. (1993). Expression and factor-dependent modulation of the interleukin-3 receptor subunits on human hematopoietic cells. Blood *82*, 752–761.
24. Manz, M.G., Miyamoto, T., Akashi, K., and Weissman, I.L. (2002). Prospective isolation of human clonogenic common myeloid progenitors. Proc. Natl. Acad. Sci. USA *99*, 11872–11877.
25. Korpelainen, E.I., Gamble, J.R., Vadas, M.A., and Lopez, A.F. (1996). IL-3 receptor expression, regulation and function in cells of the vasculature. Immunol. Cell Biol. *74*, 1–7.
26. Brizzi, M.F., Garbarino, G., Rossi, P.R., Pagliardi, G.L., Arduino, C., Avanzi, G.C., and Pegoraro, L. (1993). Interleukin 3 stimulates proliferation and triggers endothelial-leukocyte adhesion molecule 1 gene activation of human endothelial cells. J. Clin. Invest. *91*, 2887–2892.
27. Gill, S., Tasian, S.K., Ruella, M., Shestova, O., Li, Y., Porter, D.L., Carroll, M., Danet-Desnoyers, G., Scholler, J., Grupp, S.A., et al. (2014). Preclinical targeting of human acute myeloid leukemia and myeloablation using chimeric antigen receptor-modified T cells. Blood *123*, 2343–2354.
28. Mardiros, A., Dos Santos, C., McDonald, T., Brown, C.E., Wang, X., Budde, L.E., Hoffman, L., Aguilar, B., Chang, W.C., Bretzlaff, W., et al. (2013). T cells expressing CD123-specific chimeric antigen receptors exhibit specific cytolytic effector functions and antitumor effects against human acute myeloid leukemia. Blood *122*, 3138–3148.
29. Al-Hussaini, M., Rettig, M.P., Ritchey, J.K., Karpova, D., Uy, G.L., Eissenberg, L.G., Gao, F., Eades, W.C., Bonvini, E., Chichili, G.R., et al. (2016). Targeting CD123 in acute myeloid leukemia using a T-cell-directed dual-affinity retargeting platform. Blood *127*, 122–131.
30. Cartellieri, M., Feldmann, A., Koristka, S., Arndt, C., Loff, S., Ehninger, A., von Bonin, M., Bejestani, E.P., Ehninger, G., and Bachmann, M.P. (2016). Switching CAR T cells on and off: a novel modular platform for retargeting of T cells to AML blasts. Blood Cancer J. *6*, e458.

31. Bachmann, D., Aliperta, R., Bergmann, R., Feldmann, A., Koristka, S., Arndt, C., Loff, S., Welzel, P., Albert, S., Kegler, A., et al. (2017). Retargeting of UniCAR T cells with an *in vivo* synthesized target module directed against CD19 positive tumor cells. Oncotarget *9*, 7487–7500.

32. Albert, S., Arndt, C., Feldmann, A., Bergmann, R., Bachmann, D., Koristka, S., Ludwig, F., Ziller-Walter, P., Kegler, A., Gärtner, S., et al. (2017). A novel nanobody-based target module for retargeting of T lymphocytes to EGFR-expressing cancer cells via the modular UniCAR platform. OncoImmunology *6*, e1287246.

33. Feldmann, A., Arndt, C., Bergmann, R., Loff, S., Cartellieri, M., Bachmann, D., Aliperta, R., Hetzenecker, M., Ludwig, F., Albert, S., et al. (2017). Retargeting of T lymphocytes to PSCA- or PSMA positive prostate cancer cells using the novel modular chimeric antigen receptor platform technology "UniCAR". Oncotarget *8*, 31368–31385.

34. Mitwasi, N., Feldmann, A., Bergmann, R., Berndt, N., Arndt, C., Koristka, S., Kegler, A., Jureczek, J., Hoffmann, A., Ehninger, A., et al. (2017). Development of novel target modules for retargeting of UniCAR T cells to GD2 positive tumor cells. Oncotarget *8*, 108584–108603.

35. Pishali Bejestani, E., Cartellieri, M., Bergmann, R., Ehninger, A., Loff, S., Kramer, M., Spehr, J., Dietrich, A., Feldmann, A., Albert, S., et al. (2017). Characterization of a switchable chimeric antigen receptor platform in a pre-clinical solid tumor model. OncoImmunology *6*, e1342909.

36. Mahnke, Y.D., Brodie, T.M., Sallusto, F., Roederer, M., and Lugli, E. (2013). The who's who of T-cell differentiation: human memory T-cell subsets. Eur. J. Immunol. *43*, 2797–2809.

37. Bonifant, C.L., Szoor, A., Torres, D., Joseph, N., Velasquez, M.P., Iwahori, K., Gaikwad, A., Nguyen, P., Arber, C., Song, X.T., et al. (2016). CD123-engager T cells as a novel immunotherapeutic for acute myeloid leukemia. Mol. Ther. *24*, 1615–1626.

38. Mardiros, A., Forman, S.J., and Budde, L.E. (2015). T cells expressing CD123 chimeric antigen receptors for treatment of acute myeloid leukemia. Curr. Opin. Hematol. *22*, 484–488.

39. Budde, L., Song, J.Y., Kim, Y., Blanchard, S., Wagner, J., Stein, A.S., Weng, L., Del Real, M., Hernandez, R., Marcucci, E., et al. (2017). Remissions of acute myeloid leukemia and blastic plasmacytoid dendritic cell neoplasm following treatment with CD123-specific CAR T cells: a first-in-human clinical trial. Blood *130* (*Suppl 1*), 811.

40. Koristka, S., Cartellieri, M., Feldmann, A., Arndt, C., Loff, S., Michalk, I., Aliperta, R., von Bonin, M., Bornhäuser, M., Ehninger, A., et al. (2014). Flexible antigen-specific redirection of human regulatory T cells via a novel universal chimeric antigen receptor system. Blood *124*, 3494.

41. Bonifant, C.L., Jackson, H.J., Brentjens, R.J., and Curran, K.J. (2016). Toxicity and management in CAR T-cell therapy. Mol. Ther. Oncolytics *3*, 16011.

42. Bonnet, D., and Dick, J.E. (1997). Human acute myeloid leukemia is organized as a hierarchy that originates from a primitive hematopoietic cell. Nat. Med. *3*, 730–737.

43. Lapidot, T., Sirard, C., Vormoor, J., Murdoch, B., Hoang, T., Caceres-Cortes, J., Minden, M., Paterson, B., Caligiuri, M.A., and Dick, J.E. (1994). A cell initiating human acute myeloid leukaemia after transplantation into SCID mice. Nature *367*, 645–648.

44. Hope, K.J., Jin, L., and Dick, J.E. (2004). Acute myeloid leukemia originates from a hierarchy of leukemic stem cell classes that differ in self-renewal capacity. Nat. Immunol. *5*, 738–743.

45. Jordan, C.T., Upchurch, D., Szilvassy, S.J., Guzman, M.L., Howard, D.S., Pettigrew, A.L., Meyerrose, T., Rossi, R., Grimes, B., Rizzieri, D.A., et al. (2000). The interleukin-3 receptor alpha chain is a unique marker for human acute myelogenous leukemia stem cells. Leukemia *14*, 1777–1784.

46. Blair, A., Hogge, D.E., and Sutherland, H.J. (1998). Most acute myeloid leukemia progenitor cells with long-term proliferative ability in vitro and in vivo have the phenotype $CD34^{+}/CD71^{-}/HLA\text{-}DR^{-}$. Blood *92*, 4325–4335.

47. Venton, G., Pérez-Alea, M., Baier, C., Fournet, G., Quash, G., Labiad, Y., Martin, G., Sanderson, F., Poullin, P., Suchon, P., et al. (2016). Aldehyde dehydrogenases inhibition eradicates leukemia stem cells while sparing normal progenitors. Blood Cancer J. *6*, e469.

48. Crews, L.A., Balaian, L., Delos Santos, N.P., Leu, H.S., Court, A.C., Lazzari, E., Sadarangani, A., Zipeto, M.A., La Clair, J.J., Villa, R., et al. (2016). RNA splicing modulation selectively impairs leukemia stem cell maintenance in secondary human AML. Cell Stem Cell *19*, 599–612.

49. Taussig, D.C., Miraki-Moud, F., Anjos-Afonso, F., Pearce, D.J., Allen, K., Ridler, C., Lillington, D., Oakervee, H., Cavenagh, J., Agrawal, S.G., et al. (2008). Anti-CD38 antibody-mediated clearance of human repopulating cells masks the heterogeneity of leukemia-initiating cells. Blood *112*, 568–575.

50. Cox, C.V., Evely, R.S., Oakhill, A., Pamphilon, D.H., Goulden, N.J., and Blair, A. (2004). Characterization of acute lymphoblastic leukemia progenitor cells. Blood *104*, 2919–2925.

51. Tettamanti, S., Marin, V., Pizzitola, I., Magnani, C.F., Giordano Attianese, G.M., Cribioli, E., Maltese, F., Galimberti, S., Lopez, A.F., Biondi, A., et al. (2013). Targeting of acute myeloid leukaemia by cytokine-induced killer cells redirected with a novel CD123-specific chimeric antigen receptor. Br. J. Haematol. *161*, 389–401.

52. Pizzitola, I., Anjos-Afonso, F., Rouault-Pierre, K., Lassailly, F., Tettamanti, S., Spinelli, O., Biondi, A., Biagi, E., and Bonnet, D. (2014). Chimeric antigen receptors against CD33/CD123 antigens efficiently target primary acute myeloid leukemia cells in vivo. Leukemia *28*, 1596–1605.

53. Tamada, K., Geng, D., Sakoda, Y., Bansal, N., Srivastava, R., Li, Z., and Davila, E. (2012). Redirecting gene-modified T cells toward various cancer types using tagged antibodies. Clin. Cancer Res. *18*, 6436–6445.

54. Rodgers, D.T., Mazagova, M., Hampton, E.N., Cao, Y., Ramadoss, N.S., Hardy, I.R., Schulman, A., Du, J., Wang, F., Singer, O., et al. (2016). Switch-mediated activation and retargeting of CAR-T cells for B-cell malignancies. Proc. Natl. Acad. Sci. USA *113*, E459–E468.

55. Ma, J.S., Kim, J.Y., Kazane, S.A., Choi, S.H., Yun, H.Y., Kim, M.S., Rodgers, D.T., Pugh, H.M., Singer, O., Sun, S.B., et al. (2016). Versatile strategy for controlling the specificity and activity of engineered T cells. Proc. Natl. Acad. Sci. USA *113*, E450–E458.

56. Chichili, G.R., Huang, L., Li, H., Burke, S., He, L., Tang, Q., Jin, L., Gorlatov, S., Ciccarone, V., Chen, F., et al. (2015). A CD3×CD123 bispecific DART for redirecting host T cells to myelogenous leukemia: preclinical activity and safety in nonhuman primates. Sci. Transl. Med. *7*, 289ra82.

57. Uy, G.L., Aldoss, I., Foster, M.C., Sallman, D.A., Sweet, K.L., Rizzieri, D.A., Sayre, P.H., Advani, A.S., Emadi, A., Wieduwilt, M.J., et al. (2019). Flotetuzumab, an investigational CD123 × CD3 bispecific Dart® protein, in salvage therapy for primary refractory and early relapsed acute myeloid leukemia (AML) patients. Blood *134* (*Suppl 1*), 733.

58. Uy, G.L., Rettig, M.P., Vey, N., Godwin, J., Foster, M.C., Rizzieri, D.A., Arellano, M.L., Topp, M.S., Huls, G., Jongen-Lavrencic, M., et al. (2018). Phase 1 cohort expansion of flotetuzumab, a CD123×CD3 bispecific DART® protein in patients with relapsed/refractory acute myeloid leukemia (AML). Blood *132* (*Suppl 1*), 764.

59. Nijmeijer, B.A., Szuhai, K., Goselink, H.M., van Schie, M.L., van der Burg, M., de Jong, D., Marijt, E.W., Ottmann, O.G., Willemze, R., and Falkenburg, J.H. (2009). Long-term culture of primary human lymphoblastic leukemia cells in the absence of serum or hematopoietic growth factors. Exp. Hematol. *37*, 376–385.

60. Koristka, S., Cartellieri, M., Arndt, C., Bippes, C.C., Feldmann, A., Michalk, I., Wiefel, K., Stamova, S., Schmitz, M., Ehninger, G., et al. (2013). Retargeting of regulatory T cells to surface-inducible autoantigen La/SS-B. J. Autoimmun. *42*, 105–116.

61. Cartellieri, M., Koristka, S., Arndt, C., Feldmann, A., Stamova, S., von Bonin, M., Töpfer, K., Krüger, T., Geib, M., Michalk, I., et al. (2014). A novel ex vivo isolation and expansion procedure for chimeric antigen receptor engrafted human T cells. PLoS ONE *9*, e93745.

62. Meinke, A., Barahmand-Pour, F., Wöhrl, S., Stoiber, D., and Decker, T. (1996). Activation of different Stat5 isoforms contributes to cell-type-restricted signaling in response to interferons. Mol. Cell. Biol. *16*, 6937–6944.

OMTO, Volume *17*

Supplemental Information

Rapidly Switchable Universal CAR-T Cells

for Treatment of CD123-Positive Leukemia

Simon Loff, Josephine Dietrich, Jan-Erik Meyer, Julia Riewaldt, Johannes Spehr, Malte von Bonin, Cordula Gründer, Mridula Swayampakula, Kristin Franke, Anja Feldmann, Michael Bachmann, Gerhard Ehninger, Armin Ehninger, and Marc Cartellieri

Figure S1

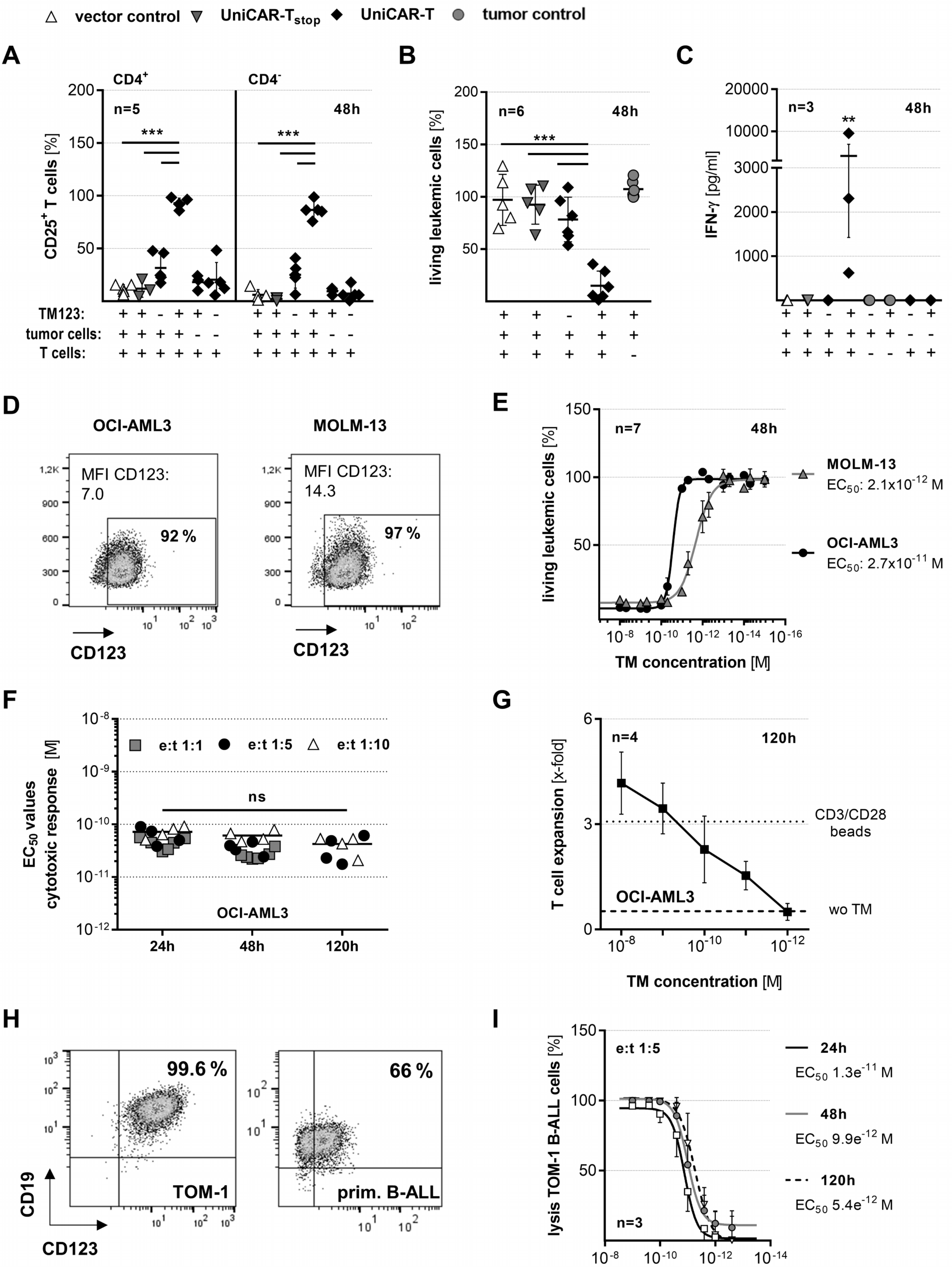
vector control
UniCAR-T$_{stop}$
UniCAR-T
tumor control
A
CD4$^+$
CD4$^-$
n=5
48h
***
CD25$^+$ T cells [%]
TM123:
tumor cells:
T cells:
B
n=6
48h
***
living leukemic cells [%]
C
n=3
48h
**
IFN-γ [pg/ml]
D
OCI-AML3
MOLM-13
MFI CD123: 7.0
92 %
MFI CD123: 14.3
97 %
CD123
E
n=7
48h
living leukemic cells [%]
TM concentration [M]
MOLM-13
EC$_{50}$: 2.1x10^{-12} M
OCI-AML3
EC$_{50}$: 2.7x10^{-11} M
F
e:t 1:1
e:t 1:5
e:t 1:10
ns
EC$_{50}$ values cytotoxic response [M]
OCI-AML3
24h
48h
120h
G
n=4
120h
T cell expansion [x-fold]
CD3/CD28 beads
OCI-AML3
wo TM
TM concentration [M]
H
99.6 %
TOM-1
66 %
prim. B-ALL
CD19
CD123
I
e:t 1:5
n=3
lysis TOM-1 B-ALL cells [%]
24h
EC$_{50}$ 1.3e^{-11} M
48h
EC$_{50}$ 9.9e^{-12} M
120h
EC$_{50}$ 5.4e^{-12} M

Figure S2

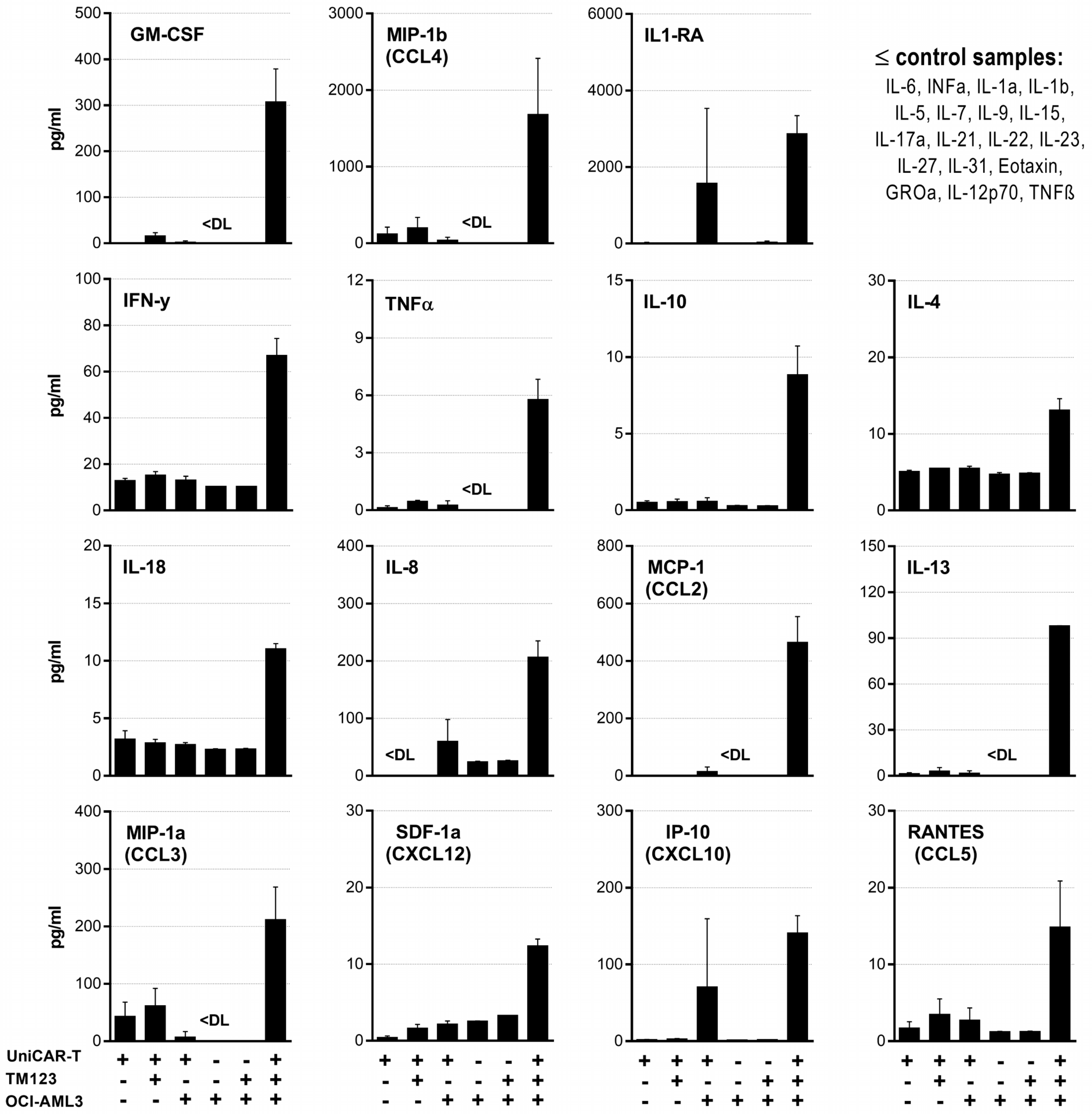

GM-CSF
MIP-1b (CCL4)
IL1-RA
≤ control samples:
IL-6, INFa, IL-1a, IL-1b, IL-5, IL-7, IL-9, IL-15, IL-17a, IL-21, IL-22, IL-23, IL-27, IL-31, Eotaxin, GROa, IL-12p70, TNFß
IFN-y
TNFα
IL-10
IL-4
IL-18
IL-8
MCP-1 (CCL2)
IL-13
MIP-1a (CCL3)
SDF-1a (CXCL12)
IP-10 (CXCL10)
RANTES (CCL5)
pg/ml
<DL
UniCAR-T
TM123
OCI-AML3

Figure S3

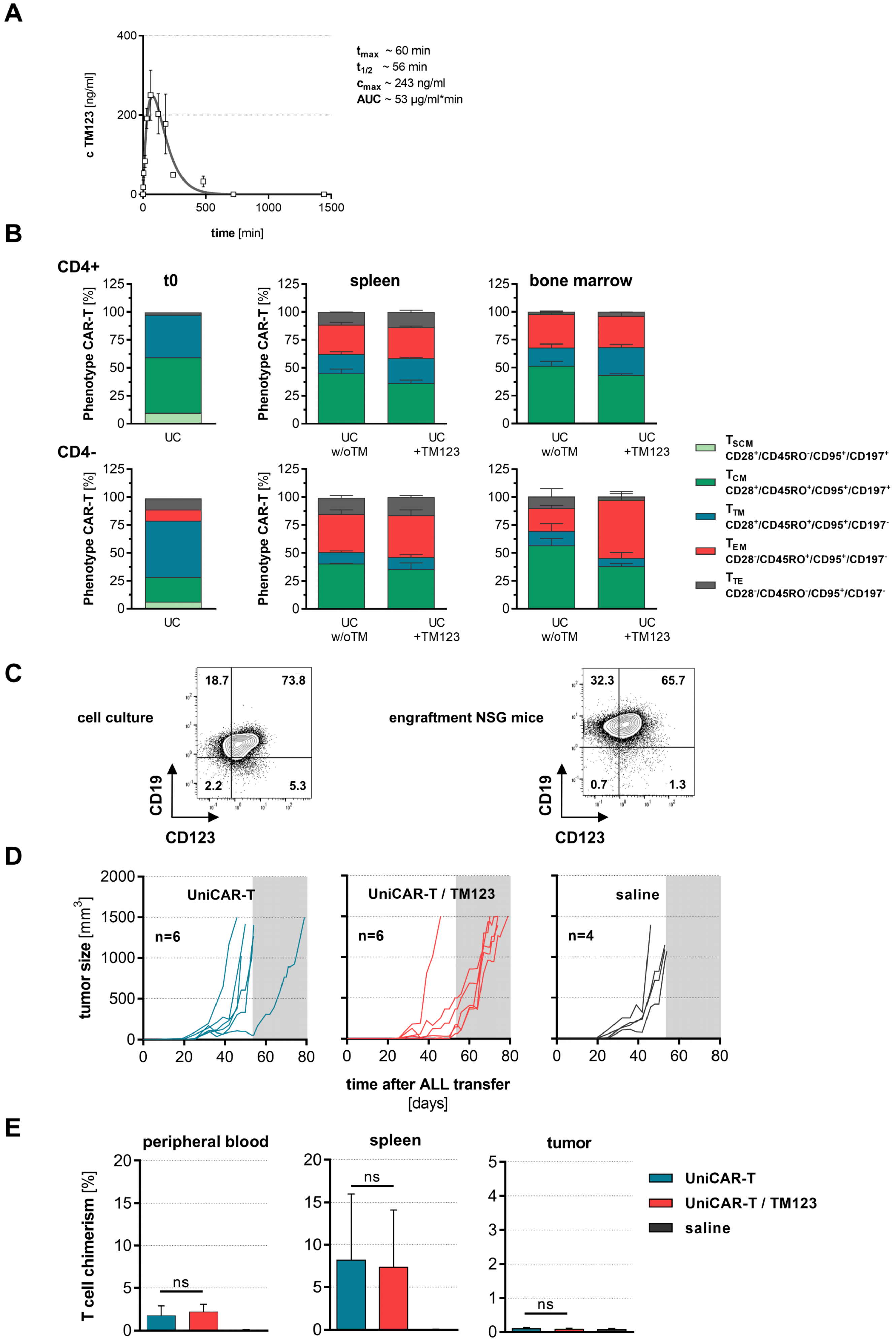
A
c TM123 [ng/ml]
time [min]
t_max ~ 60 min
t_1/2 ~ 56 min
c_max ~ 243 ng/ml
AUC ~ 53 µg/ml*min
B
CD4+
CD4-
t0
spleen
bone marrow
Phenotype CAR-T [%]
UC
UC w/oTM
UC +TM123
T_SCM CD28+/CD45RO-/CD95+/CD197+
T_CM CD28+/CD45RO+/CD95+/CD197+
T_TM CD28+/CD45RO+/CD95+/CD197-
T_EM CD28-/CD45RO+/CD95+/CD197-
T_TE CD28-/CD45RO-/CD95+/CD197-
C
cell culture
18.7
73.8
2.2
5.3
engraftment NSG mice
32.3
65.7
0.7
1.3
CD19
CD123
D
tumor size [mm3]
UniCAR-T
UniCAR-T / TM123
saline
n=6
n=6
n=4
time after ALL transfer [days]
E
peripheral blood
spleen
tumor
T cell chimerism [%]
ns
UniCAR-T
UniCAR-T / TM123
saline

Figure S4

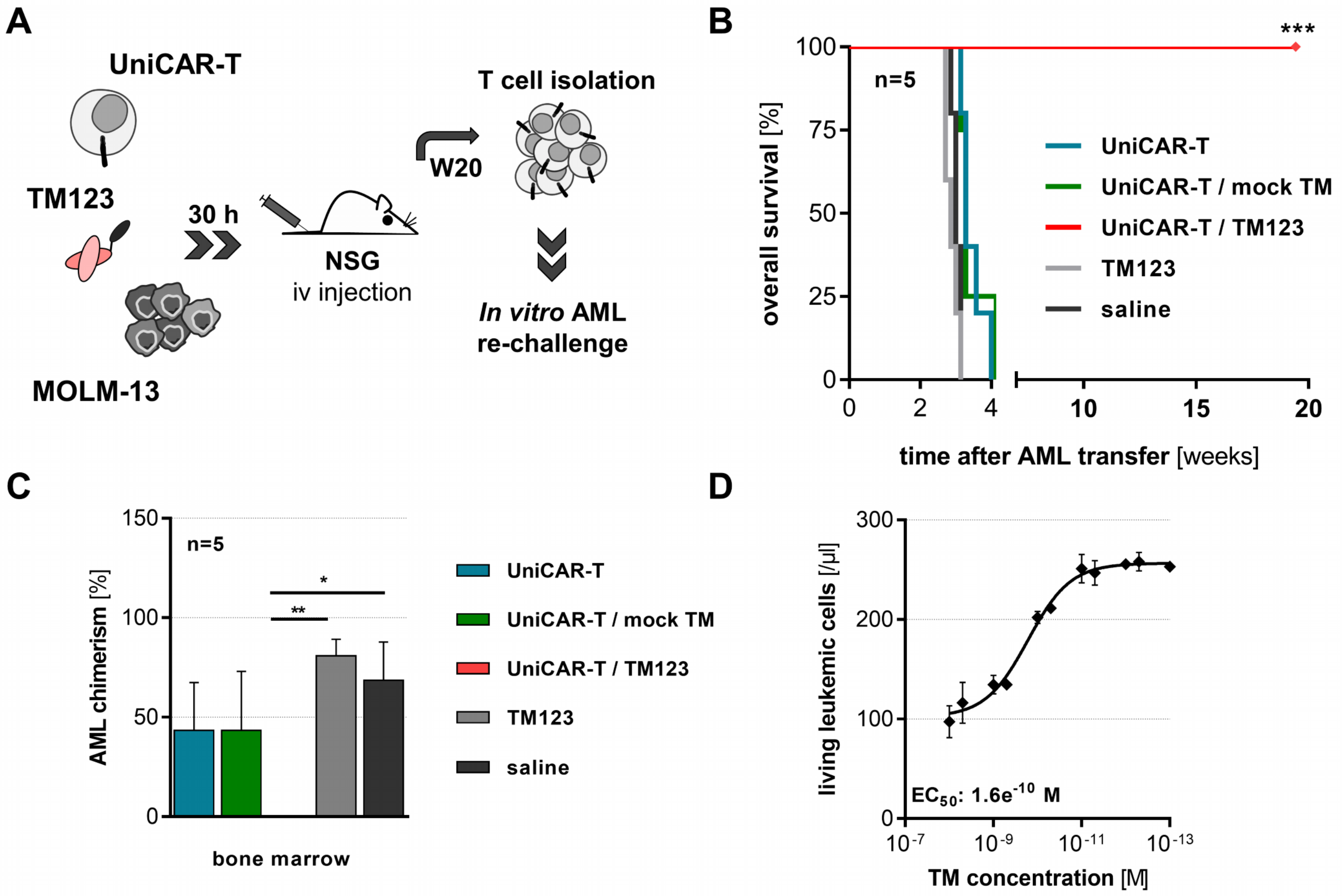
A
UniCAR-T
TM123
MOLM-13
30 h
NSG
iv injection
W20
T cell isolation
In vitro AML
re-challenge
B
***
n=5
overall survival [%]
UniCAR-T
UniCAR-T / mock TM
UniCAR-T / TM123
TM123
saline
time after AML transfer [weeks]
C
n=5
AML chimerism [%]
**
*
bone marrow
D
living leukemic cells [/µl]
EC50: 1.6e-10 M
TM concentration [M]

Figure S5

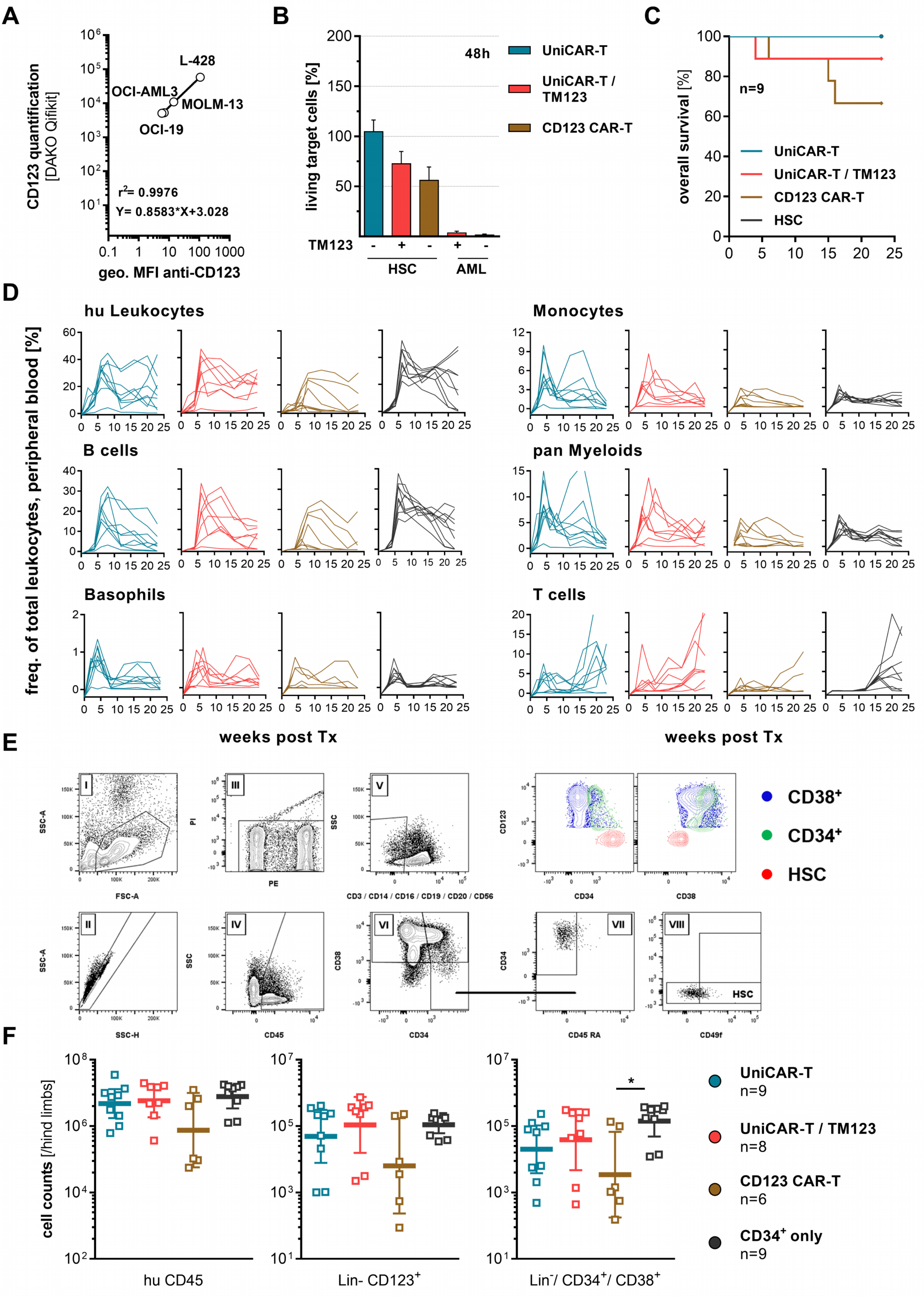

A
CD123 quantification [DAKO Qifikit]
geo. MFI anti-CD123
L-428
OCI-AML3
MOLM-13
OCI-19
r²= 0.9976
Y= 0.8583*X+3.028
B
living target cells [%]
48h
TM123
HSC
AML
UniCAR-T
UniCAR-T / TM123
CD123 CAR-T
C
overall survival [%]
n=9
UniCAR-T
UniCAR-T / TM123
CD123 CAR-T
HSC
D
freq. of total leukocytes, peripheral blood [%]
hu Leukocytes
Monocytes
B cells
pan Myeloids
Basophils
T cells
weeks post Tx
weeks post Tx
E
SSC-A
FSC-A
PI
PE
SSC
CD3 / CD14 / CD16 / CD19 / CD20 / CD56
CD123
CD34
CD38
CD38⁺
CD34⁺
HSC
SSC-H
CD45
CD45 RA
CD49f
F
cell counts [/hind limbs]
hu CD45
Lin- CD123⁺
Lin⁻/ CD34⁺/ CD38⁺
*
UniCAR-T n=9
UniCAR-T / TM123 n=8
CD123 CAR-T n=6
CD34⁺ only n=9

Supplemental figure legends

Figure S1. Anti-leukemic response of TM123-redirected UniCAR-T. Human T cells were genetically engineered to express either functional UniCARs, signalling-deficient UniCARs ($UniCAR_{stop}$) or enhanced green fluorescent protein only (vector control). (**A**) Induction of the activation marker CD25 on $CD4^+$ and $CD4^-$ UniCAR-T ($2x10^4$) cultivated with MOLM-13 at an e:t ratio of 1:1 was determined by flow cytometry after 48 h (mean ± SD). (**B**) Lysis of MOLM-13 cells after 48 h in presence of TM123 normalized to controls lacking UniCAR-T (mean ± SD). (**C**) Release of IFN-γ after 48 h co-cultivation of UniCAR-T with MOLM-13 (mean ± SEM). (**D**) CD123 expression on the AML cell lines OCI-AML3 and MOLM-13. (**E**) EC_{50} values for effective cytotoxic responses of TM123-activated UniCAR-T against OCI-AML3 and MOLM-13 after 48 h cultivation (mean ± SD). (**F**) Cytotoxic responses against OCI-AML-3 ($5x10^4$) mediated by decreasing numbers of UniCAR-T (mean). (**G**) Expansion of UniCAR-T after 120 h cultivation with OCI-AML3 cells at e:t 1:10. UniCAR-induced T cell expansion was compared to expansion-rates upon stimulation with CD3 / CD28 T cell activator beads (mean ± SEM). (**H**) CD123 expression on the B-ALL cell line TOM-1 and primary B-ALL blasts. (**I**) EC_{50} values for effective cytotoxic responses of TM123-activated UniCAR-T against TOM-1 B-ALL cells after 24h, 48h and 120h cultivation (mean ± SD). Data was obtained for indicated numbers of T cell donors. Statistical significance for (**A**), (**B**), and (**F**) was assessed by one-way analysis of variance (ANOVA) with *post hoc* Dunnet's multiple comparison test (* $P \leq 0.05$, ** $P \leq 0.01$, *** $P \leq 0.001$, ns, non-significant).

Figure S2. Cytokine profile of TM123-redirected UniCAR-T after cultivation with OCI-AML3. UniCAR-T were cultured together with OCI-AML3 cells in an effector to target cell ratio (e:t) of 1:1 in the presence or absence of 10 nM TM123 for 48 h. Cytokine release was quantified from cell-free culture supernatants using a cytokine-chemokine-34plex analysis. Data was obtained for n=2 independent T cell donors (mean ± deviation).

Figure S3. *In vivo* efficacy of TM123-redirected UniCAR-T against systemic and extramedullary acute leukemia. (**A**) In order to determine half-life of TM123, NSG mice were injected intraperitoneally (ip) with 1000 ng/g TM123, normalized to the bodyweight of mice. Peripheral blood samples were taken at indicated time points. Concentration of TM123 was determined via in-house ELISA.[30] (**B**) Analysis of the UniCAR-T phenotype after the TM123 treatment cycle in the systemic MOLM-13 xenograft model was performed via staining of CD3, CD4, CD28, CD45RO, CD95 and CD197. Resulting T cell subsets were classified as stem cell memory T cells (T_{SCM}), central memory T cells (T_{CM}), transitional memory T cells (T_{TM}), effector memory T cells (T_{EM}) and terminal effector T cells (T_{TE}) (mean ± SEM) according to Mahnke et al. 2013.[36] (**C**) Subcutaneously (sc) transplanted primary B-ALL blasts retain stable CD123 surface expression in NSG mice. (**D**) Tumor progression of sc transplanted primary B-ALL monitored for all individual mice. Highlighted regions indicate TM123-free period post tumor-therapy. (**E**) Analysis of $CD3^+$ T cell frequencies in peripheral blood, spleen and tumor tissue at the individual end points (mean ± SEM). Statistical significance was assessed by nonparametric one-way analysis of variance (ANOVA; Kruskal-Wallis test) with *post hoc* Dunn's multiple comparison test (* $P \leq 0.05$, ns, non-significant).

Figure S4. TM123-redirected UniCAR-T eliminate acute leukemia in an AML-Winn type assay. (**A**) UniCAR-T ($1x10^5$) were pre-incubated with MOLM-13 cells at an effector to target cell (e:t) ratio of 1:1 with or without TM123 for 30 h prior intravenous (iv) transplantation into immunodeficient NSG mice (Winn-assay). Subsequently, isolated T cells were re-challenged with MOLM-13 *in vitro*. (**B**) Overall survival was monitored for 20 weeks. (**C**) Significant reduction of leukemic cells in BM after *ex vivo* pre-incubation (Winn-assay) of MOLM-13 with UniCAR-T and 5 nM TM123. (**D**) After 20 weeks of engraftment in NSG mice purified T cells from spleens were re-challenged with MOLM-13 cells *in vitro*. Anti-tumor response of TM123-redirected UniCAR-T was determined after 48 h at e:t 1:1. Statistical significance was assessed by (**B**) Kaplan-Meier method with log-rank (Mantel-Cox) or by (**C**) nonparametric one-way analysis of variance (ANOVA; Kruskal-Wallis test) with *post hoc* Dunn's multiple comparison test (* $P \leq 0.05$, ** $P \leq 0.01$, *** $P \leq 0.001$).

Figure S5. Analysis of human cell engraftment in peripheral blood and bone marrow of humanized NSG mice. (**A**) CD123 was quantified on representative cell lines using DAKO Qifikit®. CD123 density on further cell lines, primary cells or patient derived samples was calculated via antigen standard curve. (**B**) Cytotoxic response of transplanted donor-specific UniCAR-T in presence or absence of 5 nM TM123 and CD123 CAR-T against isolated $CD34^+$ hematopoietic progenitors or MOLM-13 cells was assessed after 48 h in parallel to the *ex vivo* incubation samples (mean ± SD). (**C**) Overall survival of humanized NSG mice. (**D**) Human leukocyte subsets in the peripheral blood (PB) of all individual recipient mice. (**E**) Gating strategy for identification of HSC-enriched cell pools in bone marrow (BM) samples. (**F**) Absolute numbers of human leukocytes and hematopoietic progenitors per hind limbs (femur + tibia). All samples were analyzed 23 weeks post transplantation. Statistical significance was assessed by parametric one-way analysis of variance (ANOVA) with *post hoc* Dunnet's multiple comparison test (* $P \leq 0.05$).
